# Precision targeting of autoantigen-specific B cells in muscle-specific tyrosine kinase myasthenia gravis with chimeric autoantibody receptor T cells

**DOI:** 10.1038/s41587-022-01637-z

**Published:** 2023-01-19

**Authors:** Sangwook Oh, Xuming Mao, Silvio Manfredo-Vieira, Jinmin Lee, Darshil Patel, Eun Jung Choi, Andrea Alvarado, Ebony Cottman-Thomas, Damian Maseda, Patricia Y. Tsao, Christoph T. Ellebrecht, Sami L. Khella, David P. Richman, Kevin C. O’Connor, Uri Herzberg, Gwendolyn K. Binder, Michael C. Milone, Samik Basu, Aimee S. Payne

**Affiliations:** 1 Department of Dermatology, Perelman School of Medicine, University of Pennsylvania, Philadelphia, PA, USA.; 2 Cabaletta Bio, Philadelphia, PA, USA.; 3 Department of Neurology, Perelman School of Medicine, University of Pennsylvania, Philadelphia, PA, USA.; 4 Department of Neurology, University of California – Davis, Davis, CA, USA.; 5 Departments of Neurology and Immunobiology, Yale School of Medicine, New Haven, CT, USA.; 6 Department of Pathology and Laboratory Medicine, Perelman School of Medicine, University of Pennsylvania, Philadelphia, PA, USA.

## Abstract

Muscle-specific tyrosine kinase myasthenia gravis (MuSK MG) is an autoimmune disease that causes life-threatening muscle weakness due to anti-MuSK autoantibodies that disrupt neuromuscular junction signaling. To avoid chronic immunosuppression from current therapies, we engineered T cells to express a MuSK chimeric autoantibody receptor with CD137-CD3ζ signaling domains (MuSK-CAART) for precision targeting of B cells expressing anti-MuSK autoantibodies. MuSK-CAART demonstrated similar efficacy as anti-CD19 chimeric antigen receptor T cells for depletion of anti-MuSK B cells and retained cytolytic activity in the presence of soluble anti-MuSK antibodies. In an experimental autoimmune MG mouse model, MuSK-CAART reduced anti-MuSK IgG without decreasing B cells or total IgG levels, reflecting MuSK-specific B cell depletion. Specific off-target interactions of MuSK-CAART were not identified in vivo, in primary human cell screens or by high-throughput human membrane proteome array. These data contributed to an investigational new drug application and phase 1 clinical study design for MuSK-CAART for the treatment of MuSK autoantibody-positive MG.

Muscle-specific tyrosine kinase myasthenia gravis (MuSK MG) is a chronic autoimmune disorder caused by MuSK autoantibodies that result in potentially life-threatening muscle weakness^1-3^. MuSK is a transmembrane receptor that interacts with lipoprotein receptor-related protein 4 (LRP4) in complex with the neuronal proteoglycan agrin^4,5^, which leads to MuSK phosphorylation and formation of high-density acetylcholine receptor (AChR) clusters that are essential for neuromuscular junction synaptic transmission^6^.

MuSK MG disease severity correlates with anti-MuSK antibody titers^7^, particularly IgG4 autoantibodies targeting the MuSK Ig1 domain, which disrupt MuSK-LRP4 interactions^8-10^. B cell depletion with rituximab results in reduction of anti-MuSK IgG relative to total IgG^11^, indicating that MuSK autoantibodies are primarily produced by short-lived plasma cells^12,13^. Disease relapse after rituximab is attributed to incomplete B cell depletion^14^, requiring repeated rituximab infusions for disease control, but chronic B cell depletion risks serious infections. Therapy should ideally eliminate only the pathogenic anti-MuSK B cells and spare healthy B cells to achieve durable remission of MuSK MG without generalized immunosuppression.

Here, we report the design and functional validation of a chimeric autoantibody receptor (CAAR) comprising the MuSK autoantigen, tethered to tandem CD137-CD3ζ signaling domains. MuSK-CAAR expression in T cells directs cytotoxicity toward B cells expressing an anti-MuSK surface autoantibody or B cell receptor (BCR). MuSK-CAAR T cell (MuSK-CAART) technology is based on a clinically approved anti-CD19 chimeric antigen receptor T cell (CART-19) therapy that has led to complete and durable remissions of B cell malignancies^15,16^. We investigated MuSK-CAART efficacy and safety in preclinical models, which support MuSK-CAART as a precision cellular immunotherapy with potential to induce complete and durable remission of MuSK MG.

## Results

### MuSK-CAAR targets disease-relevant anti-MuSK B cell epitopes

**Fig. 1 ∣:**
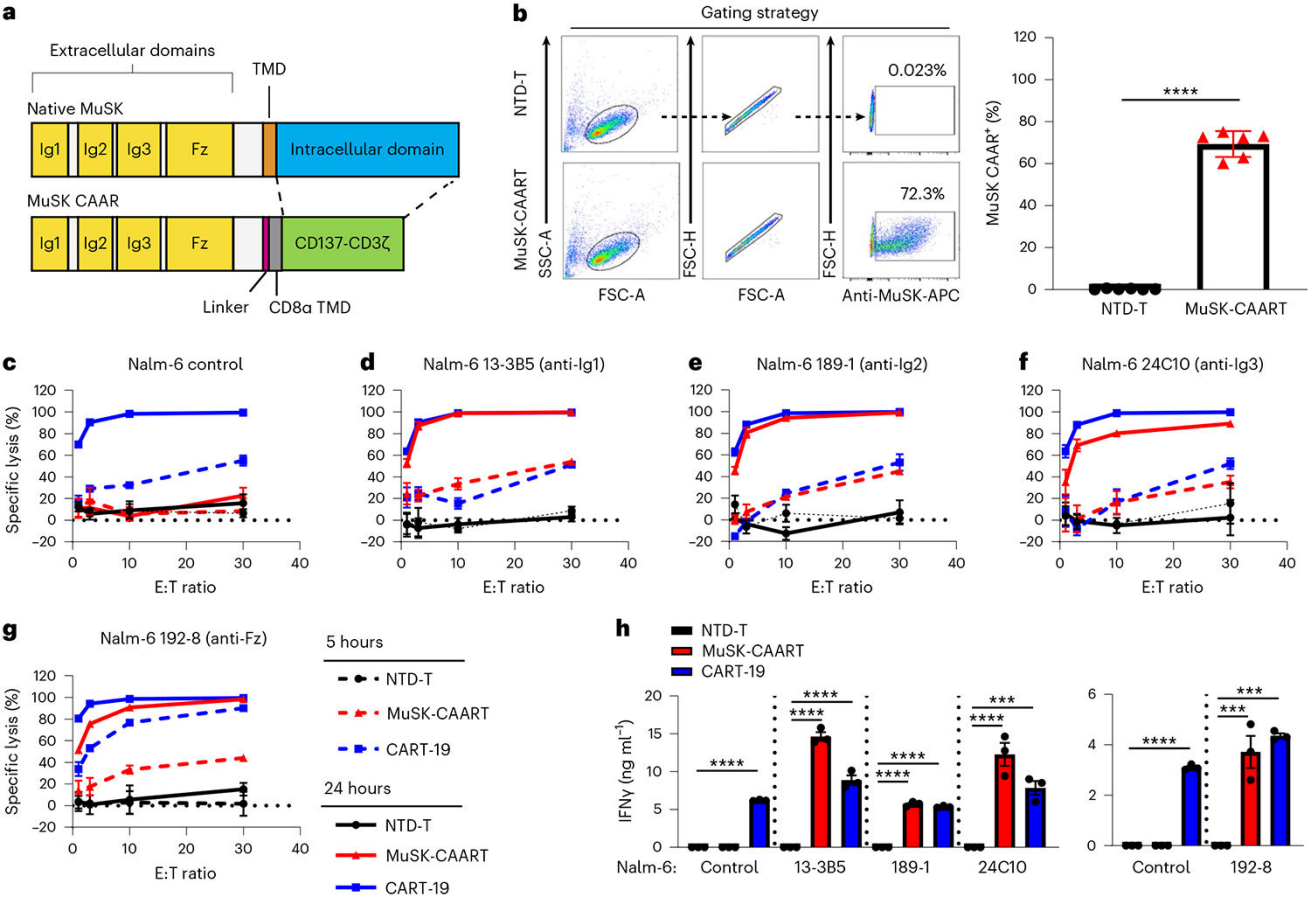
MuSK-CAAR expression on primary human T cells directs specific cytolysis of anti-MuSK B cells that target unique epitopes. **a**, Native MuSK is a transmembrane tyrosine kinase whose ectodomain comprises three immunoglobulin-like (Ig1–Ig3) and frizzled-like (Fz) domains. MuSK-CAAR comprises the native MuSK ectodomain, followed by a glycine/serine-rich linker, CD8α transmembrane domain (TMD) and CD137-CD3ζ intracellular costimulatory and activation domains. **b**, Primary human T cells were transduced with MuSK-CAAR lentivirus or NTD-T, and MuSK-CAAR expression was detected using anti-MuSK 4A3 or 189-1 antibody. MuSK-CAAR^+^ transduction efficiency in six different donor T cell batches (NTD-T and MuSK-CAART). Error bars indicate mean ± s.d. Unpaired *t*-test (two-tailed). **c–g**, Cytolysis of wild-type (control) Nalm-6 cells (**c**) and Nalm-6 anti-MuSK target cells 13-3B5/anti-Ig1 (**d**), 189-1/anti-Ig2 (**e**), 24C10/anti-Ig3 (**f**) and 192-8/anti-Fz (**g**) was measured using a luciferase-based cytotoxicity assay at 5 h (dashed line) and 24 h (solid line) after coculture with NTD-T (black), MuSK-CAART (red) and CART-19 (blue). The effector to target (E:T) ratio is based on total T cell number. **h**, Human IFNγ was measured in NTD-T, MuSK-CAART or CART-19 coculture supernatants (10:1 E:T, 24 h, experiments run using different T cell batches are shown in separate plots). One-way analysis of variance (ANOVA) with the Holm–Sidak test for multiple comparisons. For **c–h**, error bars indicate mean ± s.d. of triplicate cocultures and are representative of 2–4 independent experiments. NS, *P* > 0.05; *P < 0.05; ***P < 0.001; ****P < 0.0001.

MuSK is a transmembrane tyrosine kinase whose ectodomain comprises three immunoglobulin-like (Ig1–Ig3) and frizzled-like (Fz) domains. An estimated 100, 58 and 23% of sera from patients with MuSK MG recognize Ig1, Ig2 and Ig3-Fz domains, respectively^8^. To target anti-MuSK B cells, we designed a MuSK-CAAR comprising the complete MuSK ectodomain, linked to CD137-CD3ζ costimulatory and activation domains, and confirmed expression on primary human T cells (Fig. 1a,b).

**Extended Data Fig. 1 ∣:**
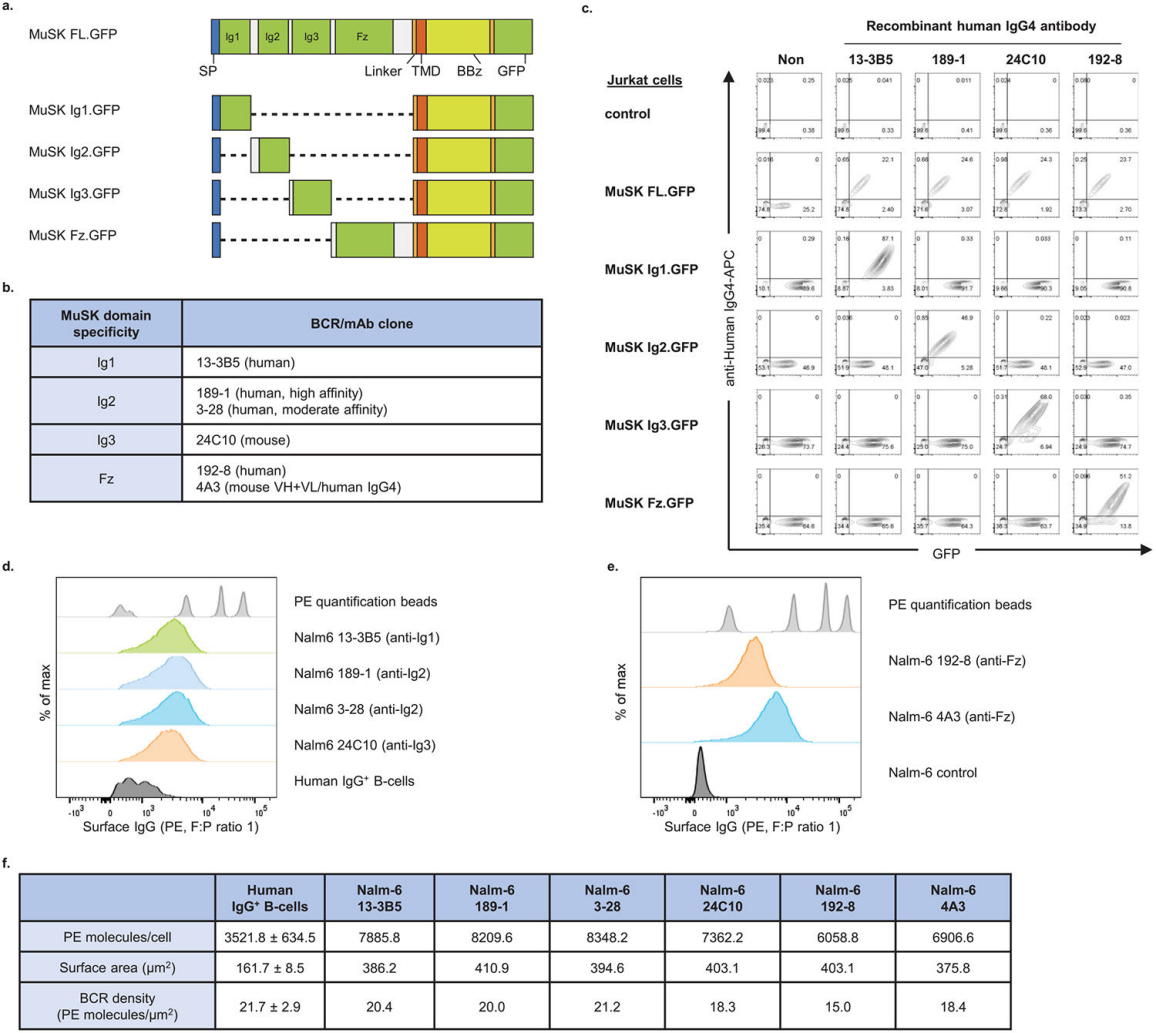
Anti-MuSK target cell characterization. **a**) Schematic diagram of individual MuSK domain CAARs with a cytoplasmic linker to green fluorescent protein (GFP), generated for epitope mapping. FL, full-length; SP, signal peptide; TMD, transmembrane domain; BBζ, CD137(4-1BB)-CD3ζ. **b**) Summary of recombinant anti-MuSK B cell receptor (BCR) or mAb specificities. **c**) MuSK domain CAAR^+^ Jurkat cells were stained with anti-MuSK mAbs to confirm the domain mapping. **d, e**) BCRs in primary human IgG^+^ B cells and Nalm-6 cells expressing each MuSK domain-specific BCR were stained with PE mouse anti-human IgG. **f**) BCR density was calculated by dividing the number of PE molecules/cell by the surface area (μm^2^). Mean ± standard deviation of IgG^+^ B-cells from three individual experiments is shown.

To validate MuSK-CAART cytotoxicity, we generated Nalm-6 B cells expressing anti-MuSK domain-specific BCRs isolated from three patients with MuSK MG or three MuSK-immunized mice. Epitope mapping and previous literature confirmed MuSK domain specificity: anti-MuSK Ig1 (13-3B5, ref. ^17^), anti-MuSK Ig2 (189-1, ref. ^18^ and 3-28, ref. ^12,18^), anti-MuSK Ig3 (24C10) and anti-MuSK Fz (4A3, refs. ^12,18^ and 192-8) (Extended Data Fig. 1a-c). Anti-MuSK BCR density on engineered Nalm-6 cells was within twofold of IgG BCR density on primary human B cells (Extended Data Fig. 1d-f).

**Extended Data Fig. 2 ∣:**
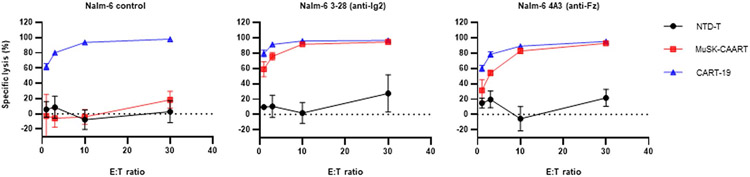
MuSK-CAAR directs specific cytolysis of anti-MuSK B-cells. NTD-T cells, anti-CD19 CART (CART-19), and MuSK-CAART were coincubated with Nalm-6 control, Nalm-6 3–28 (anti-MuSK Ig2), and Nalm-6 4A3 (anti-MuSK Fz) cell lines at indicated E:T ratios. Cytotoxicity was evaluated at 24 hours using a luciferase-based assay. Error bars indicate mean ± standard deviation of triplicates.

MuSK-CAART demonstrated specific lysis of Nalm-6 cells targeting each MuSK domain, but not control Nalm-6 cells (Fig. 1c-g and Extended Data Fig. 2), with increased specific cytotoxicity at 24 versus 5 hours of coculture. Donor-matched nontransduced (NTD) T cells (NTD-T) showed no cytotoxicity. IFNγ was detected in supernatants of MuSK-CAART cocultured with anti-MuSK Nalm-6 but not control Nalm-6 cells (Fig. 1h).

### Anti-MuSK Abs have varying effects on MuSK-CAART activity

**Extended Data Fig. 3 ∣:**
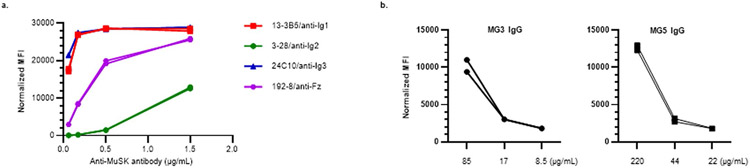
Relative titers of anti-MuSK mAbs and MG IgG. Anti-MuSK antibody titer was evaluated using a Luminex-based assay. Recombinant anti-MuSK mAbs (**a**) or purified IgG from two MuSK MG patients (MG3 and MG5) (**b**) were incubated with MuSK ectodomain-coupled microspheres and stained with anti-human IgG-biotin. Mean fluorescence intensity (MFI) was normalized per 50 beads. Error bars indicate mean ± SEM of duplicates.

**Extended Data Fig. 4 ∣:**
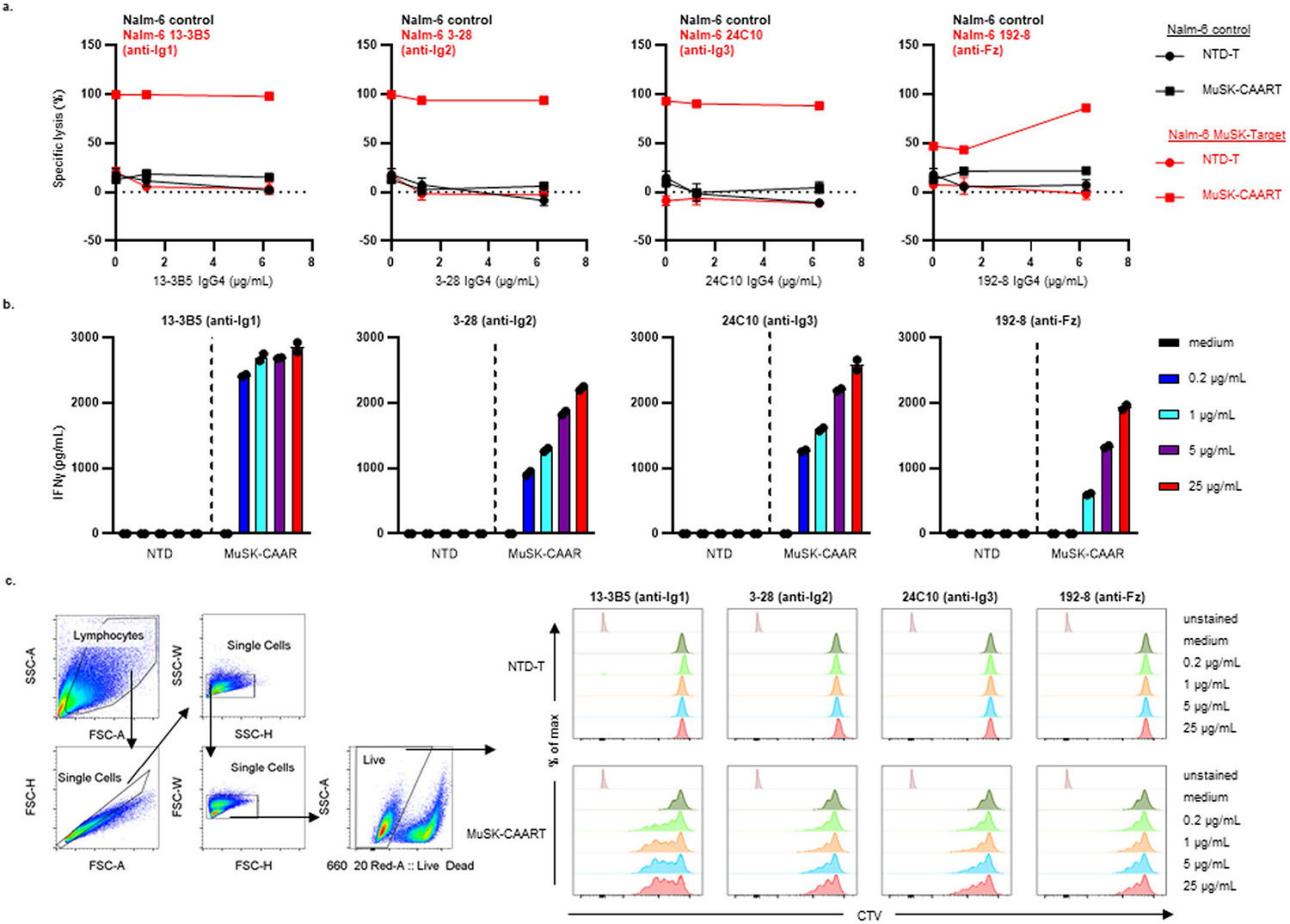
Evaluation of soluble anti-MuSK monoclonal antibody effects on MuSK-CAART cytotoxicity, IFNγ production, and proliferation. **a**) Non-transduced T cells (NTD-T) or MuSK-CAART were co-incubated with Nalm-6 control or individual MuSK domain-specific Nalm-6 target cells at an E:T ratio of 10:1 in the absence (0 μg/mL) or presence of matching soluble anti-MuSK IgG4 mAb at 1.25 or 6.25 μg/mL, in a total of 10 mg/mL normal human IgG. Cytotoxicity was evaluated at 24 hours using a luciferase-based assay. Error bars indicate mean ± standard deviation of triplicates. **b**) NTD-T or MuSK-CAART cells were incubated with each anti-MuSK IgG4 mAb (0.2, 1, 5, or 25 μg/mL) and human IFNγ was quantitated by ELISA in cell culture supernatants after 24 hours. Error bars indicate mean ± standard deviation of triplicates. **c**) Proliferation of NTD-T (top) and MuSK-CAART (bottom) was evaluated 96 hours after the addition of the indicated anti-MuSK mAbs using Cell Trace Violet (CTV) cellular labeling dye dilution by flow cytometry. (a-c) Representative plots from two (b,c) or three (a) individual experiments using different donor T-cells are shown.

**Fig. 2 ∣:**
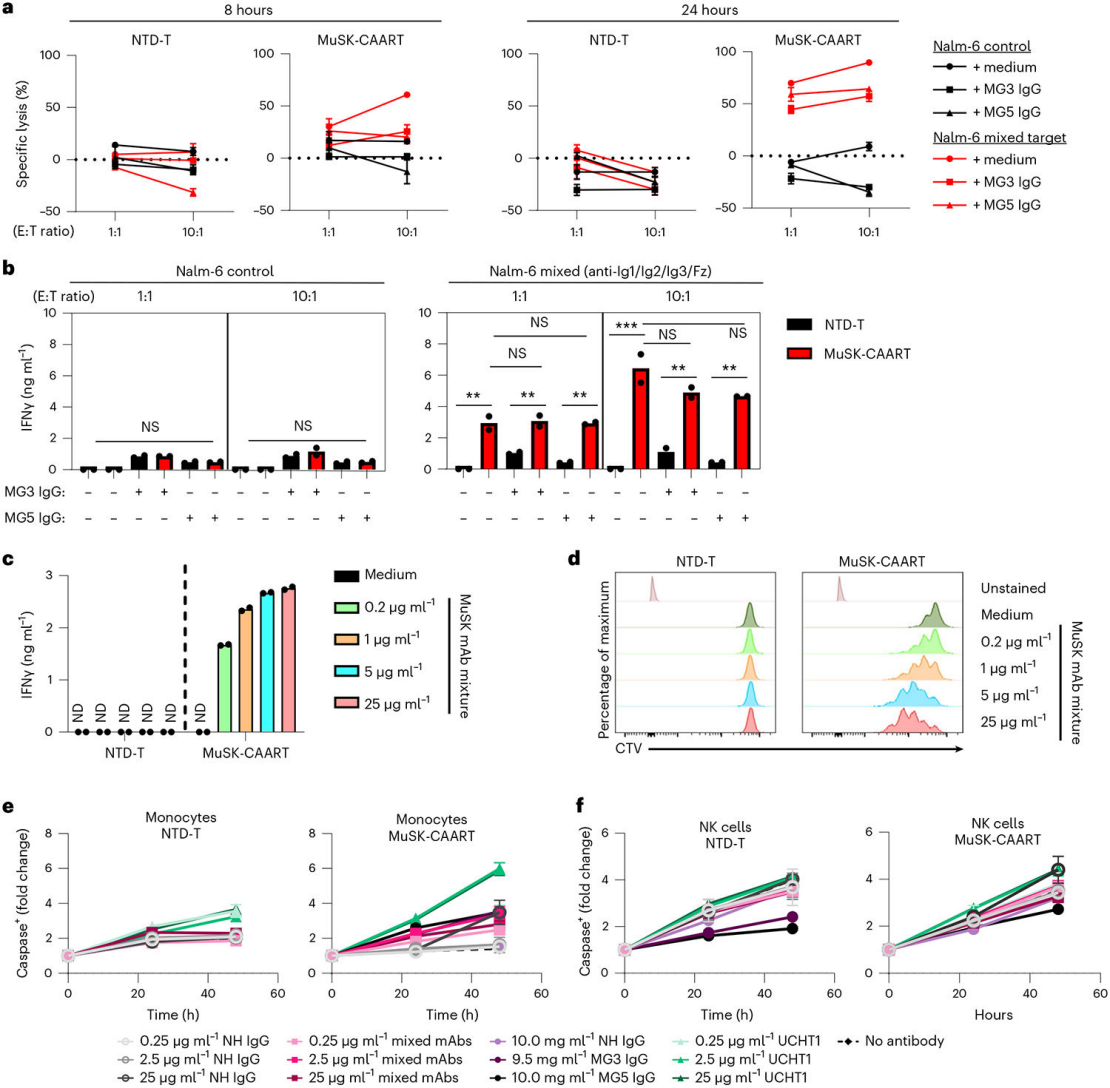
Evaluation of soluble anti-MuSK antibody effects on MuSK-CAART cytotoxicity, IFNγ production and proliferation. **a**, NTD-T and MuSK-CAART were coincubated with Nalm-6 control or mixed target cells (13-3B5/3-28/24C10/192-8 (anti-Ig1/Ig2/Ig3/Fz)) at 1:1 or 10:1 E:T ratios in the presence of purified IgGs (10 mg ml^−1^) from two patients with MuSK MG (MG3 and MG5; details in Methods) or medium alone. Cytotoxicity was evaluated at 8 and 24 h using a luciferase-based cytotoxicity assay. Error bars indicate mean ± s.d. of triplicates. **b**, Human IFNγ was measured in NTD-T or MuSK-CAART coculture supernatants (24 h) in two independent experiments. Two-way ANOVA with Tukey’s test for multiple comparisons: NS, *P* > 0.05; **P < 0.01; ***P < 0.001. **c,d**, NTD-T and MuSK-CAART were incubated with an equimolar mixture of anti-MuSK IgG4 mAbs (13-3B5/3-28/24C10/192-8 (anti-Ig1/Ig2/Ig3/Fz), total mAb concentration shown) and human IFNγ was quantitated by ELISA after 24 h in duplicated samples (**c**), or proliferation of NTD-T and MuSK-CAART was evaluated using CTV dye dilution by flow cytometry at 96 h (**d**). Representative flow plots from two individual experiments are shown. **e,f**, NTD-T or MuSK-CAART were coincubated with monocytes (**e**) or NK cells (**f**) at a 5:1 E:T ratio in the presence of normal human IgG, mixed anti-MuSK mAbs (13-3B5/3-28/192-8 (anti-Ig1/Ig2/Fz), total mAb concentration shown), purified polyclonal IgG from plasma from patients with MuSK MG (MG3 and MG5) or an anti-CD3 positive-control mAb (clone UCHT1) for 48 h. Monocyte/NK cell death was detected by incorporation of caspase-3/7 dye over time. Fold change of caspase^+^ cells relative to the 0 hour timepoint is plotted. Error bars indicate mean ± s.d. of triplicates.

MuSK autoantibodies might block MuSK-CAAR engagement with anti-MuSK BCRs, but they could also potentiate cytotoxicity by activating MuSK-CAART. Anti-MuSK IgG concentrations in sera from patients with MuSK MG range from 0.5 to 49.5 nM (0.16–7.4 μg ml^−1^), assuming one antibody bound per MuSK molecule^11,19,20^. To determine soluble anti-MuSK antibody effects on MuSK-CAART activity, we performed cytotoxicity assays in the presence of a physiologic concentration (10 mg ml^−1^) of polyclonal IgG from patients with MG, or individual or mixed monoclonal antibody (mAb), at concentrations within or exceeding the expected range for autoantigen-specific IgG (0.2–25 μg ml^−1^). Relative binding of MuSK IgG from patients MG3 and MG5 and anti-MuSK IgG4 mAbs appear in Extended Data Fig. 3. MuSK-CAART cytotoxicity against mixed Nalm-6 target cells (13-3B5/3-28/24C10/192-8 (anti-Ig1/Ig2/Ig3/Fz)) was partly inhibited by polyclonal IgG from patients with MuSK MG, although specific cytotoxicity generally increased with higher effector to target ratios and longer coincubation (Fig. 2a). MuSK-CAART and NTD-T produced similarly low levels of IFNγ when cultured with Nalm-6 control and IgG from patients with MuSK MG (Fig. 2b, left panel), whereas a significant increase in IFNγ production was observed in MuSK-CAART relative to NTD-T when cocultured with anti-MuSK Nalm-6 cells (Fig. 2b, right panel, black versus red bars). IgG from patients with MG did not significantly affect IFNγ levels, although a trend toward lower levels was observed (Fig. 2b, right panel, red bars). Coincubation of MuSK-CAART with monoclonal Nalm-6 cells and matching soluble anti-MuSK mAb showed similar results, except 192-8/anti-Fz IgG4 potentiated MuSK-CAART cytotoxicity at higher concentrations (Extended Data Fig. 4a).

To determine direct effects of anti-MuSK antibodies on MuSK-CAART, MuSK-CAART was incubated with anti-MuSK mAbs (13-3B5(anti-Ig1)/3-28(anti-Ig2)/24C10(anti-Ig3)/192-8 (anti-Fz)). Anti-MuSK mAbs, mixed or individually, induced IFNγ production and MuSK-CAART proliferation in a concentration-related manner (Fig. 2c,d and Extended Data Fig. 4b,c).

To evaluate whether Fc-gamma receptors (FcγRs) or neonatal Fc-receptor (FcRn) mediates indirect lysis by MuSK-CAART after binding anti-MuSK antibodies, primary human monocytes (which express high-affinity FcγR/CD64, CD32 and FcRn)^21^ and natural killer (NK) cells (which express low-affinity FcγR/CD16)^21^ were cocultured with MuSK-CAART and normal human IgG, mixed anti-MuSK IgG4 mAbs, purified plasma IgG from patients with MuSK MG or anti-CD3 positive-control antibody (Fig. 2e,f). After coincubation with MuSK-CAART or NTD-T in the presence of anti-MuSK IgG4 mAbs or purified plasma from IgG from patients with MG, caspase-3/7-positive monocytes or NK cells were similar to counts observed in the presence of normal human IgG and less than those observed with anti-CD3 positive-control antibody (UCHT1).

### BCR and CD19-targeted lysis show similar in vivo efficacy

**Extended Data Fig. 5 ∣:**
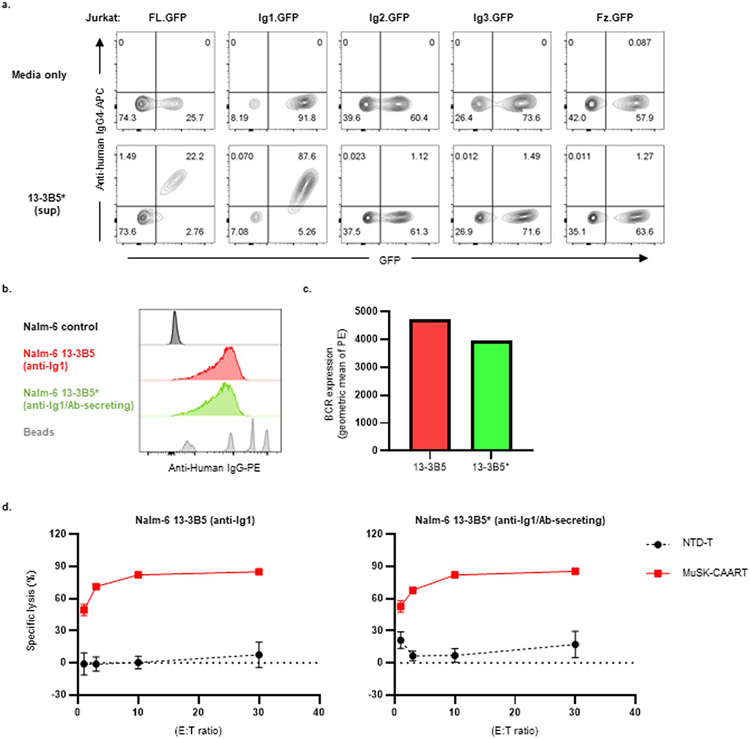
Generation and validation of 13-3B5* antibody-secreting Nalm-6 cells. **a**) Nalm-6 13-3B5* anti-Ig1 antibody-secreting cells were generated by transducing soluble 13-3B5/anti-Ig1 antibody heavy chain plasmid into Nalm-6 13-3B5 BCR–expressing cells. Jurkat cells expressing individual MuSK extracellular domains linked with GFP were stained with cell-culture supernatants from Nalm-6 13-3B5* for epitope mapping. Soluble 13-3B5 antibody binding was detected using anti-human IgG4-APC. **b, c**) Nalm-6 13-3B5 (red) and Nalm-6 13-3B5* (green) were stained with PE-conjugated mouse anti-human IgG to quantify BCR density. The mean fluorescence intensity of BCR expression is shown by histogram (b) and bar graph (c). **d**) Nalm-6 13-3B5 or Nalm-6 13-3B5* cells were co-incubated with either NTD-T or MuSK-CAART cells at indicated E:T ratios for 8 hours. MuSK-CAART cytotoxicity was measured using a luciferase-based assay. Error bars indicate mean ± standard deviation of triplicates.

We next evaluated whether anti-MuSK BCR-targeted cytolysis demonstrates comparable efficacy as CD19-targeted cytolysis in eliminating anti-MuSK B cells in vivo, using a well-characterized nod scid gamma (NSG) Nalm-6 xenograft model. NSG mice were engrafted with mixed luciferase-expressing 13-3B5/3-28/24C10/192-8 or 4A3 Nalm-6 cells (anti-Ig1/Ig2/Ig3/Fz), followed by treatment with MuSK-CAART, NTD-T or CART-19. In parallel experiments to evaluate the potential neutralizing effect of anti-MuSK IgG on MuSK-CAART in vivo, NSG mice were engrafted with 13-3B5/anti-Ig1 or 13-3B5* antibody-secreting Nalm-6 cells (described in Methods and Extended Data Fig. 5).

**Extended Data Fig. 6 ∣:**
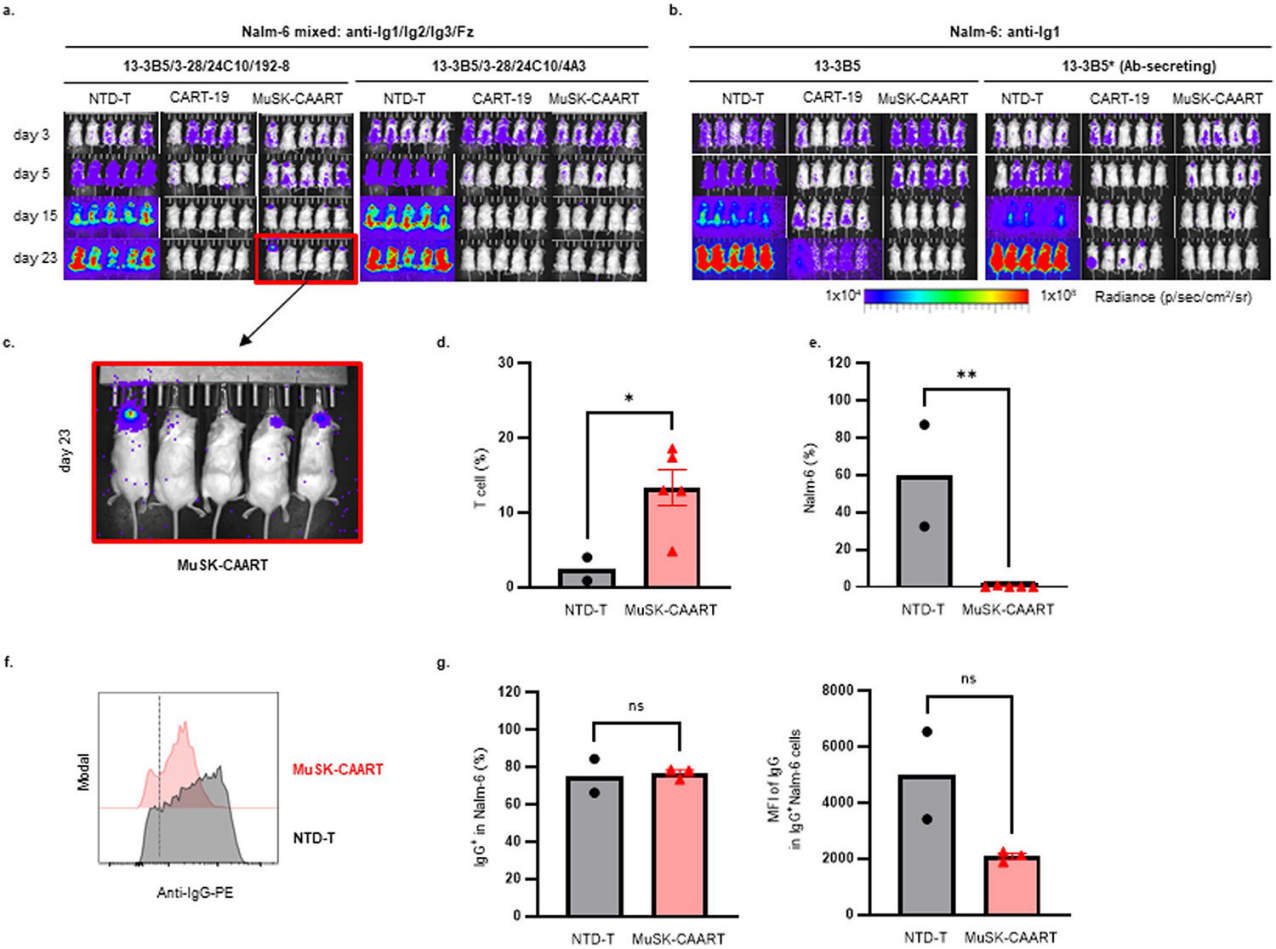
Evaluation of Nalm-6 outgrowth in a subset of MuSK-CAART-treated mice. **a, b**) Bioluminescence images from Fig. 3a, b. c) Enlarged bioluminescence image from (a, red box). **d, e**) T-cell and Nalm-6 cell percentage in the cranial bone marrow in NTD-T treated mice (n = 2) and MuSK-CAART treated mice (n = 5) were analyzed by flow cytometry. **f**) Representative plot showing the mean fluorescence intensity (MFI) of IgG BCR expression in residual Nalm-6 cells (dotted line indicates cutoff for positive surface IgG expression). **g**) Graphs indicate the percent of residual Nalm-6 cells that are IgG BCR + and the MFI of IgG BCR expression in residual Nalm-6 cells in NTD-T (n = 2) and MuSK-CAART (n = 3) treated mice. Error bars indicate mean ± SEM. Unpaired *t*-test (two-tailed): ns, p > 0.05; *, p < 0.05; **, p < 0.01.

**Fig. 3 ∣:**
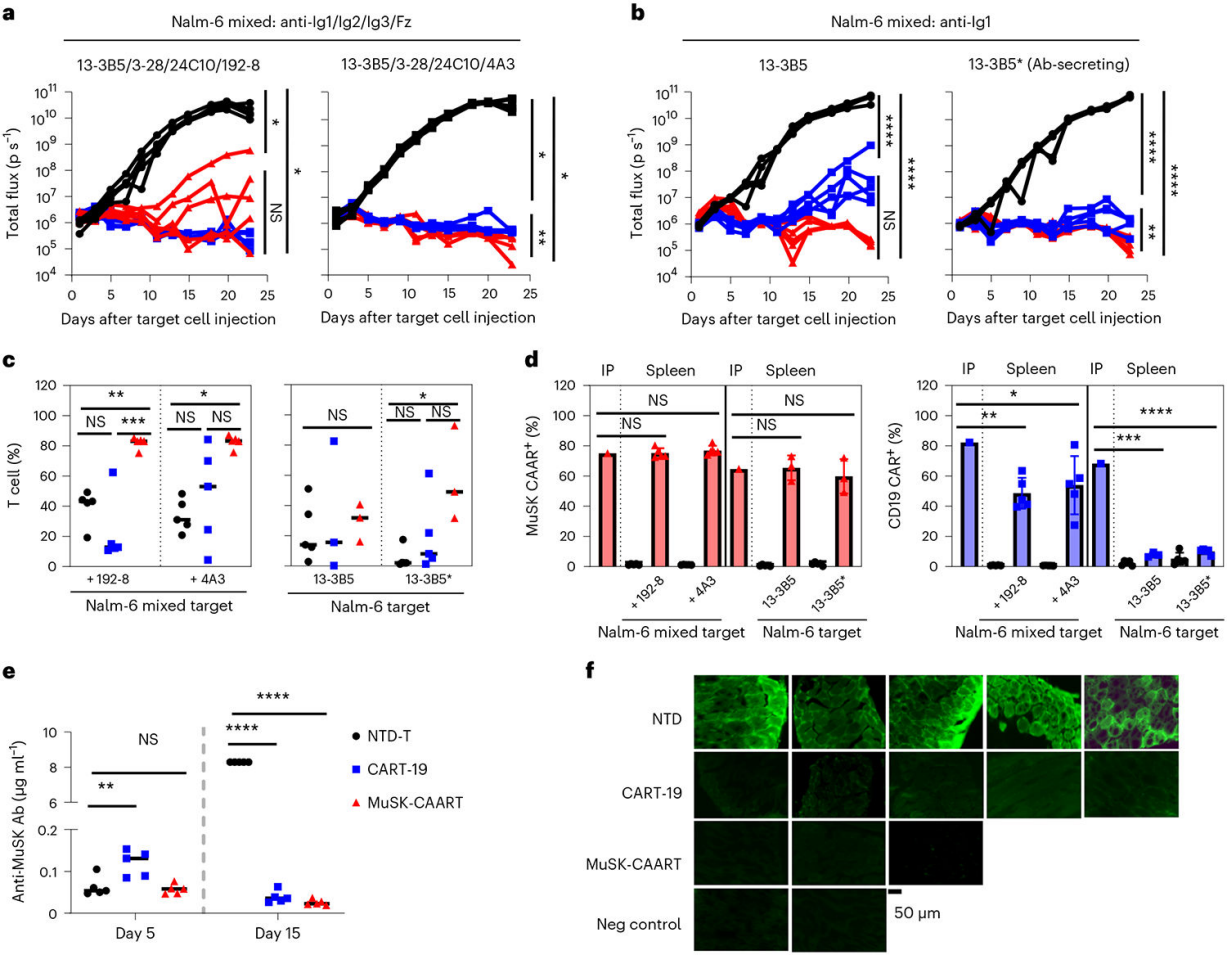
Targeting of anti-MuSK B cells through the BCR with MuSK-CAART demonstrates comparable efficacy as anti-CD19 CAR-mediated cytolysis, in the presence or absence of soluble anti-MuSK antibody. **a,b**, Total flux (p s^−1^, photons per second) after injection of 1:1:1:1 mixed 13-3B5/3-28/24C10/192-8 or 13-3B5/3-28/24C10/4A3 (anti-Ig1/Ig2/Ig3/Fz) (**a**), 13-3B5 (anti-Ig1) or 13-3B5* (anti-Ig1, antibody-secreting) Nalm-6 cells (**b**), followed 4 days later by treatment with 1 × 10^7^ NTD-T (black, *n* = 5), CART-19 (blue, *n* = 5) or MuSK-CAART (red, *n* = 5). Bioluminescence images appear in Extended Data Fig. 6a. One-way ANOVA with the Holm–Sidak test for multiple comparisons, day 23. **c,d**, Splenocytes were analyzed on days 24 or 25 after target cell injection for CD3^+^ T cell frequency (**c**) (median values are indicated; Kruskal–Wallis test with Dunnett’s test for multiple comparisons), and percentage of MuSK-CAAR^+^ and anti-CD19 CAR^+^ T cells relative to the infusion product (IP) (**d**). Error bars indicate mean ± s.e.m. One-sample *t*-test. Two MuSK-CAART-treated mice in Nalm-6 13-3B5/13-3B5* and two CART-19-treated mice in Nalm-6 13-3B5 experiments that were used for longterm follow-up were excluded from analysis. **e**, Anti-MuSK antibody titer in blood samples was measured on days 5 and 15 after target cell injection and quantitated relative to a 13-3B5 IgG4 mAb standard. Two-way ANOVA with Dunnett’s test for multiple comparisons. **f**, Direct immunofluorescence analysis of diaphragm muscle harvested on day 25, stained with antihuman IgG to detect 13-3B5 IgG4 binding to MuSK on the muscle cell surface. Diaphragms from 13-3B5/NTD-T-treated mice served as a negative control. Scale bar, 50 μm. Representative images are shown from two independent staining experiments. NS, *P* > 0.05; *P < 0.05; **P < 0.01; ***P < 0.001; ****P < 0.0001.

Bioluminescence imaging from all four experiments indicated that CART-19 and MuSK-CAART significantly reduced Nalm-6 outgrowth relative to NTD-T (Fig. 3a,b), although recurrence of bioluminescence flux signal was observed in a subset of MuSK-CAART-treated and CART-19-treated mice. Nalm-6 recurrence in CART-19-treated mice engrafted with Nalm-6 13-3B5 cells was not due to loss of T cells, as T cells were detectable in most CART-19-treated mice (Fig. 3c). Similarly, Nalm-6 outgrowth in MuSK-CAART-treated mice engrafted with mixed Nalm-6 (192-8) cells was not due to failure of MuSK-CAART trafficking to cranial bone marrow (Extended Data Fig. 6c,d). In the subset of mice (*n* = 3) with residual Nalm-6 target cells in cranial bone marrow, the percentage of IgG BCR^+^ Nalm-6 cells was comparable between MuSK-CAART- and NTD-T-treated mice, but IgG BCR expression level was significantly reduced in MuSK-CAART-treated mice (Extended Data Fig. 6c-g). The low BCR density of Nalm-6 192-8, which is greater than two standard deviations lower than the mean density on primary human IgG^+^ B cells (Extended Data Fig. 1f), may explain Nalm-6 recurrence, since low target antigen density negatively affects CART cytotoxic efficacy^22^. In MuSK-CAART-treated mice engrafted with mixed Nalm-6 (4A3) cells, all target cells were eliminated, comparable to CART-19-treated mice, and no target cell recurrence was observed (Fig. 3a, right).

Higher MuSK-CAART percentages were observed in mixed Nalm-6 and 13-3B5*-engrafted mice (Fig. 3c), potentially due to soluble mAb-induced expansion or proliferation of MuSK-CAART in vivo after target cell encounter. MuSK-CAAR expression remained stable in T cells isolated from spleen (Fig. 3d) compared to MuSK-CAART infusion product. However, anti-CD19 CAR expression was lower in spleen T cells (Fig. 3d) compared to CART-19 infusion product, which may explain Nalm-6 recurrence in CART-19-treated mice.

In 13-3B5*-xenografted mice, anti-MuSK antibody titer increased in mice treated with NTD-T from day 5 to 15, whereas titers in CART-19- and MuSK-CAART-treated mice were significantly reduced compared to NTD-T-treated mice by day 15, 11 days after T cell injection (Fig. 3e). Anti-MuSK IgG binding to diaphragm muscle cells in CART-19- and MuSK-CAART-treated mice was also reduced compared to NTD-T-treated mice (Fig. 3f).

Taken together, these data indicate that MuSK-CAART can target MuSK Ig1/Ig2/Ig3/Fz domain-specific cells with comparable efficacy to CART-19.

### MuSK-CAART efficacy in a syngeneic MuSK EAMG model

**Extended Data Fig. 7 ∣:**
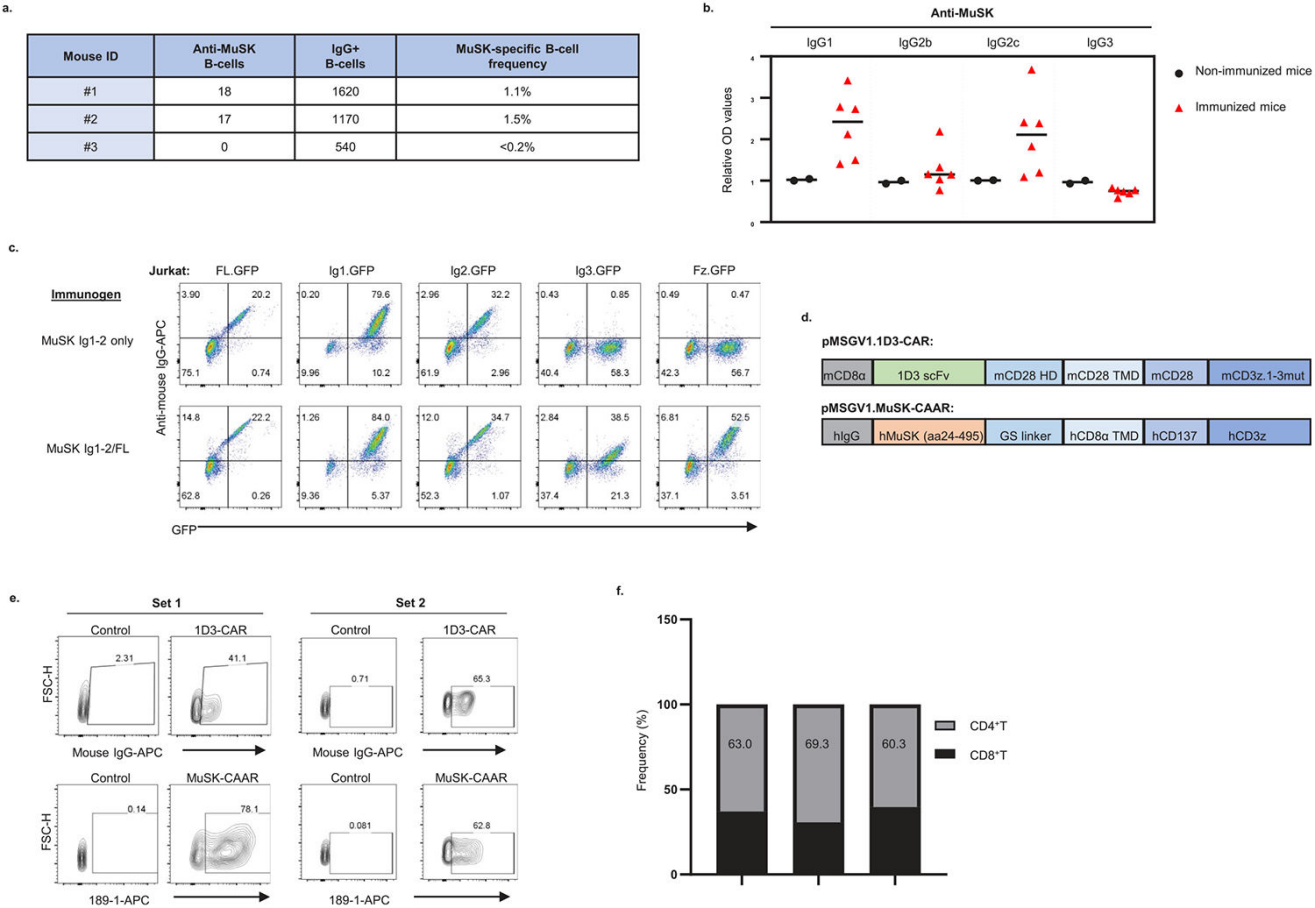
Immunologic characterization of the MuSK EAMG syngeneic MuSK-CAART treatment model. **a**) Splenocytes were harvested from select MuSK-immunized mice, and purified B-cells were evaluated using a MuSK-specific and total IgG B-cell ELISpot assay to quantitate the MuSK-specific B-cell frequency. Dots from duplicated wells were summarized after normalization with seeded cells (dots per 100,000 cells). MuSK-specific B-cell frequency is calculated by dividing anti-MuSK B-cells by total IgG^+^ B-cells. **b**) IgG subclasses of anti-MuSK antibodies were detected in sera from MuSK-immunized mice in reference to sera from negative controls (non-immunized C57BL/6 and Rag2IL2rγ-deficient mice) and median value was plotted. **c**) Epitope mapping of serum from a mouse immunized and boosted with MuSK Ig1-Ig2, or MuSK Ig1-Ig2 followed by MuSK full length (FL) boost, using Jurkat CAAR T-cells expressing individual MuSK domain CAARs linked to GFP. Representative FACS plots are shown from three independent experiments. **d**) Schematic of pMSGV1-1D3-CAR (mouse CD8α signal peptide, 1D3 anti-mouse CD19 single chain variable fragment (scFv), mouse CD28 hinge (HD)/transmembrane domain (TMD)/costimulatory domain, and a mouse CD3ζ.1-3mut domain to confer enhanced persistence. Schematic of pMSGV1-MuSK-CAAR (human MuSK extracellular domains (amino acids 24-495), glycine-serine (GS) linker, hCD8α TMD, and human CD137-CD3ζ). **e, f**) Transduction efficiency of 1D3-CAR and MuSK-CAAR in primary mouse T-cells (Set 1 and Set 2) and **g**) percentage of CD4^+^/CD8^+^ T-cells in Set 1 was evaluated on day 4 after T-cell activation (one day prior to injection).

To determine whether MuSK-CAART demonstrates efficacy in an immunocompetent mouse model that recapitulates features of autoimmune pathophysiology, including rare anti-MuSK B cells and polyclonal autoantibodies, we evaluated MuSK-CAART efficacy in a syngeneic MuSK experimental autoimmune myasthenia gravis (EAMG) model. Classic MuSK EAMG models involve immunization of mice with rat MuSK^23,24^; use of human MuSK for EAMG induction is associated with variable symptom onset, although symptomatic mice demonstrate reduction in miniature endplate potentials and postsynaptic AChR density, consistent with an MG phenotype^25-27^. After day 0 immunization and day 26 boost of C57BL/6 (CD45.2^+^) mice with human MuSK ectodomain fragments, anti-MuSK IgG B cells comprised <0.2–1.5% of total splenocyte IgG B cells gathered on day 34 (Extended Data Fig. 7a). Serum anti-MuSK antibodies were predominantly of the IgG1 and IgG2c subclasses (Extended Data Fig. 7b). Anti-MuSK antibodies targeted all four MuSK domains (Extended Data Fig. 7c). Immunized mice were treated on day 35 with murine CD45.1^+^ T cells expressing human MuSK-CAAR, a 1D3/anti-CD19 CAR with modified murine CD28-CD3ζ signaling domains that confer greater in vivo efficacy as a positive control^28^ or NTD-T (Extended Data Fig. 7d). Mice were dosed with equivalent numbers of CAR/CAAR-transduced cells and matching numbers of NTD-T (Extended Data Fig. 7e). 1D3-CART and MuSK-CAART exhibited similar CD4:CD8 T cell ratios (Extended Data Fig. 7f).

**Fig. 4 ∣:**
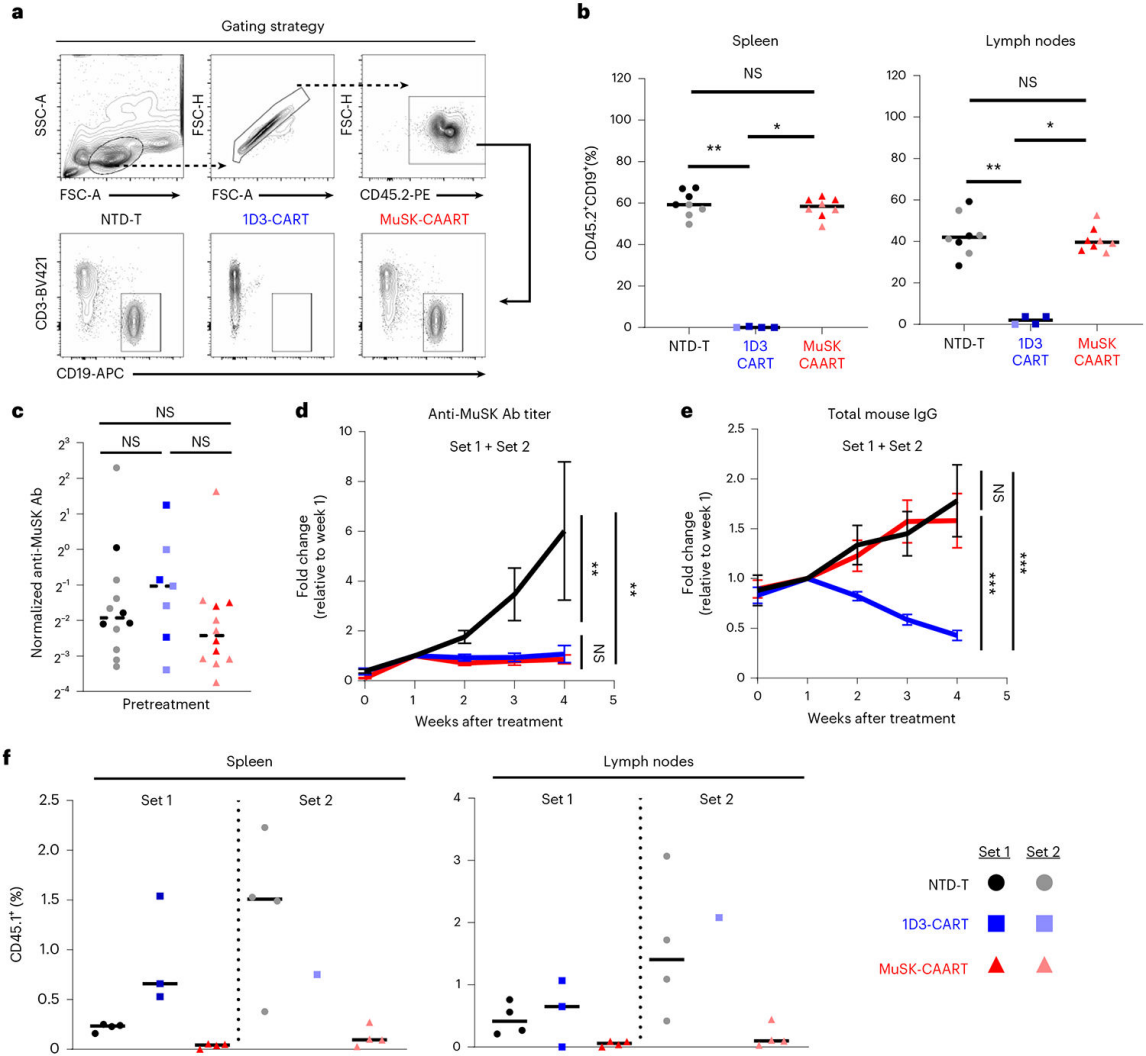
MuSK-CAART reduces anti-MuSK IgG but not total IgG or B cell counts in a syngeneic MuSK EAMG model. CD45.2^+^ C57BL/6J mice were immunized with MuSK Ig1-2 protein (30 μg in complete Freund’s adjuvant) on day 0 and boosted with MuSK Ig1-Fz protein (30 μg in incomplete Freund’s adjuvant) on day 26. Results of two independent experiments are shown (total numbers NTD-T, *n* = 12, 1D3-CART, *n* = 8, MuSK-CAART, *n* = 12). Equivalent numbers of transduced CD45.1^+^ T cells or a matching number of NTD CD45.1^+^ cells were injected on day 35. **a,b**, Host B cells (CD45.2^+^CD3^−^CD19^+^) were analyzed in spleen and lymph nodes at days 49–63 (2–4 weeks after treatment). Representative flow plot (**a**) and the frequency of CD45.2^+^CD19^+^ B cells in the spleen and lymph nodes (**b**) are shown. Kruskal-Wallis test with Dunnett’s test for multiple comparisons. **c**, Anti-MuSK antibody titer was measured in individual mouse blood samples drawn on the day of treatment, normalized to 4A3 mouse antihuman MuSK mAb standard μg ml^−1^). Kruskal–Wallis with Dunnett’s test for multiple comparisons. **d,e**, Anti-MuSK antibody titer and total mouse IgG were measured in mouse blood samples drawn weekly after treatment. Graphs indicate fold change of anti-MuSK antibody titer (**d**) or total mouse IgG (**e**) relative to week 1 after treatment (NTD-T, *n* = 8; 1D3-CART, *n* = 7 (**d**) and *n* = 6 (**e**); MuSK-CAART, *n* = 8 to include all mice with longitudinal samples through week 4 after treatment; one 1D3-CART mouse was excluded in **e** due to low blood sample volume precluding analysis). Error bars indicate mean ± s.e.m. Multiple linear regression-coefficient test for difference between the slopes. **f**, Frequency of CD45.1^+^ T cells were analyzed in the spleen and lymph nodes on day 49–63. Statistical analysis was not performed since absolute number of CD45.1^+^ T cells varied among treatment groups to achieve the same transduced cell dose in set 1. NS, *P* > 0.05; *P < 0.05; **P < 0.01; ***P < 0.001.

After treatment, few to no B cells were detected in the spleen and lymph nodes of 1D3-CART-treated mice, whereas NTD-T- and MuSK-CAART-treated mice showed comparable B cell frequency (Fig. 4b). Anti-MuSK antibody titer was comparable across treatment groups before treatment (Fig. 4c) and progressively increased in NTD-T-treated mice, whereas titers in MuSK-CAART- and 1D3-CART-treated mice significantly decreased, starting 1 week after injection (Fig. 4d). Unlike 1D3-CART, which significantly decreased total serum IgG, MuSK-CAART did not reduce total serum IgG levels relative to NTD-T-treated mice (Fig. 4e). CD45.1^+^ T cells were detected in spleen and lymph nodes with relatively lower percentages of engrafted T cells in MuSK-CAART-treated mice, and higher T cell percentages in 1D3-CART-treated mice, the latter in part due to the twofold higher number of T cells injected in set 1, in addition to the rarity of anti-MuSK B cells relative to CD19-expressing B cells in immunized mice and/or the differing signaling and costimulatory domains of MuSK-CAAR and 1D3-CAR (Fig. 4f and Extended Data Fig. 7a,d).

These data indicate that in an EAMG model with rare anti-MuSK target B cells and circulating anti-MuSK antibodies, MuSK-CAART treatment results in antigen-specific IgG depletion without total B cell depletion and does not require previous lymphodepletion for therapeutic effect.

### Specific off-target toxicity by MuSK-CAART was not observed

To evaluate for potential off-target interactions of MuSK-CAART, we performed (1) comprehensive organ histopathology in an NSG xenograft model, (2) screening of a high-throughput human membrane proteome array (MPA), (3) primary human cell screens and (4) targeted investigations based on potential MuSK-interacting proteins.

**Fig. 5 ∣:**
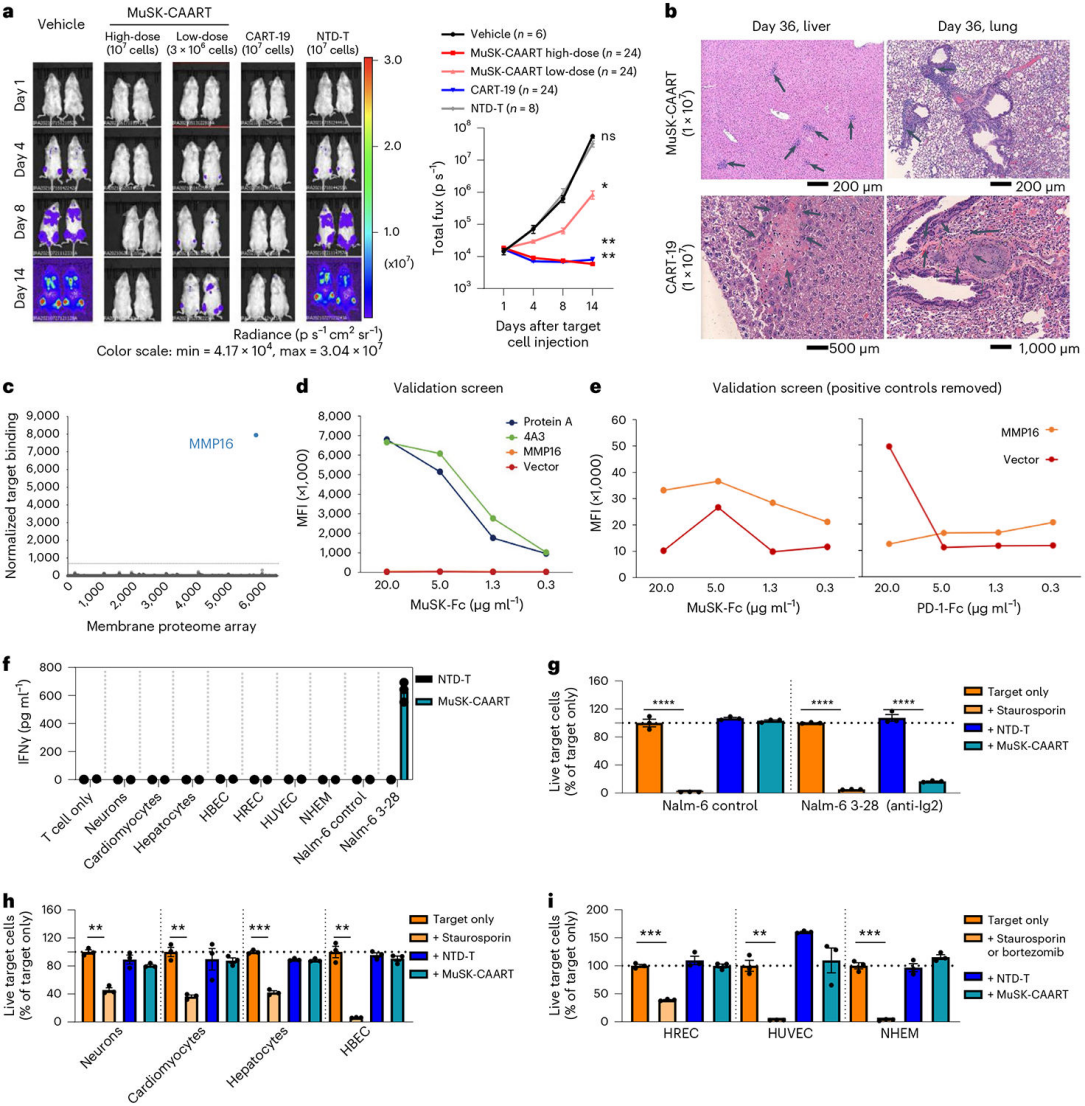
Off-target cytotoxic interactions of MuSK-CAART were not identified in mouse tissue or using human MPAs. **a**, Representative bioluminescence images from the MuSK-CAART biodistribution study in mice injected with 3-28/anti-Ig2 Nalm-6 cells, then treated with vehicle only (*n* = 6), NTD-T (*n* = 8), CART-19 (*n* = 24) or MuSK-CAART (high- and low-dose, *n* = 24 per each dose). Graph indicates total bioluminescence flux for all mice in each treatment group. Error bars indicate mean ± s.e.m. One-way ANOVA with the Holm–Sidak test for multiple comparisons, day 14. **b**, Example images from liver and lung on day 36 after treatment (MuSK-CAART, *n* = 8; CART-19, *n* = 8). Liver and lung sections from high-dose MuSK-CAART-treated mice show lymphocytic infiltration without cytotoxic effect (black arrows). CART-19-treated mice demonstrated focal hepatocellular necrosis and focal pulmonary thrombus (black arrows). **c**, Human MPA screened with MuSK-Fc protein identified a potential binding signal with MMP16. **d**, MuSK-Fc binding to MMP16 demonstrated low mean fluorescence intensity (MFI) relative to protein A and anti-MuSK 4A3 positive controls in validation screening (MMP16 curve overlaps with vector control). A representative graph from two validation screens is shown in **e. e**, Positive controls are removed in validation screens shown in **d** and the *y* axis is rescaled. One of two validation screens confirmed MuSK-Fc binding to MMP16, defined as MFI at least twofold higher than isotype (PD-1-Fc) control at two or more concentrations. **f–i**, Cytotoxicity of MuSK-CAART from two donor T cell batches was measured after coincubation for 24 hours (5:1 E:T ratio) with Nalm-6 wild-type (negative control), Nalm-6 3-28/anti-Ig2 (positive control) or seven primary human cell types from each of two different donors. Representative results are shown. IFNγ production was not detected in MuSK-CAART cocultures with primary human cells (**f**). Viability was analyzed using high-content imaging analysis (HCA) for Nalm-6 control and anti-MuSK 3-28 cells (**g**) and human-derived cells (**h**) or by flow cytometry (**i**) at 24 hours, using staurosporin or bortezomib as toxicity controls. Error bars indicate mean ± s.d. of triplicates (**f–i**). Multiple *t*-test (two-tailed), Holm–Sidak correction for multiple comparisons. NS, *P* > 0.05; *P < 0.05; **P < 0.01; ***P < 0.001; ****P < 0.0001.

MuSK-CAART biodistribution was evaluated in 146 NSG mice allocated to 19 groups, injected with 1 × 10^6^ 3-28 Nalm-6 or no target cells, followed by treatment with vehicle, 1 × 10^7^ NTD-T, 1 × 10^7^ CART-19 or 3 × 10^6^–1 × 10^7^ MuSK-CAART donor-matched cells. Representative bioluminescence images and graphs of total bioluminescence flux indicate control of Nalm-6 cell outgrowth in CART-19 and high-dose MuSK-CAART-treated mice, and delayed outgrowth in low-dose MuSK-CAART-treated mice relative to vehicle and NTD-T-treated mice (Fig. 5a). Comprehensive organ histopathologic analysis indicated lymphocytic infiltration in multiple organs, most notably at late timepoints after injection of MuSK-CAART or CART-19 in mice with target cells. Liver and lung sections from high-dose MuSK-CAART-treated mice show lymphocytic infiltration without cytotoxic effect, which could represent target cells or engraftment of the human T cell product (Fig. 5b). CART-19-treated mice demonstrated focal hepatocellular necrosis in some liver sections, consistent with xenogeneic graft-versus-host disease (xGVHD), and focal pulmonary thrombus consisting of histocytes and fibrosis, attributed to the intravenous (i.v.) route of injection (Fig. 5b). Specific off-target cytotoxic effects of MuSK-CAART relative to CART-19-treated mice were not observed.

**Extended Data Fig. 8 ∣:**
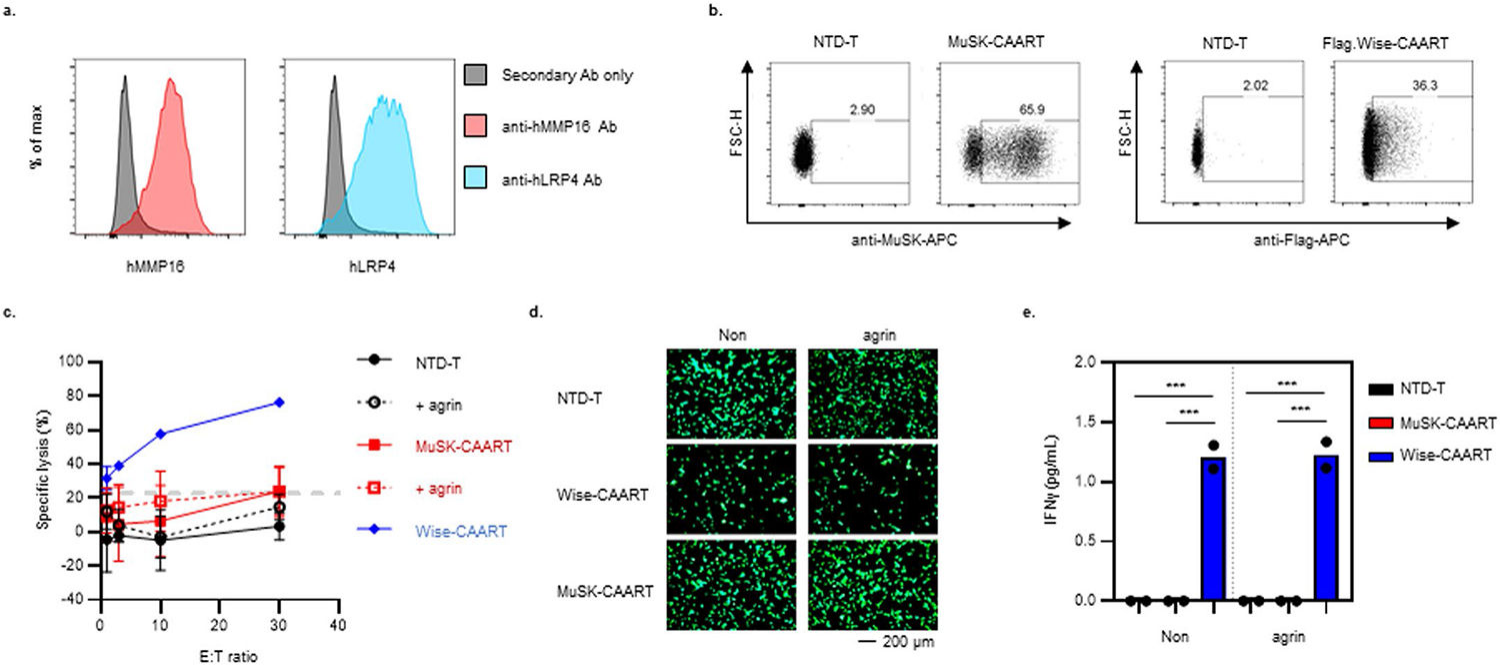
Off-target cytotoxicity of MuSK-CAART against MMP16^+^LRP4^+^ U87-MG cells was not detected. **a**) LRP4 (blue) and MMP16 (red) expression in U87-MG cells was confirmed using flow cytometry. **b**) MuSK-CAAR and Flag-tagged Wise-CAAR expression were confirmed in primary human T-cells. **c**) Luciferase^+^ U87-MG cells were co-incubated with human T-cells at the indicated E:T ratio in the presence (dashed line) or absence (solid line) of agrin. Cytotoxicity was measured using a luciferase-based killing assay. Error bars indicate mean ± standard deviation from two individual experiments (NTD-T, n = 5; NTD-T+ agrin, n = 4; MuSK-CAART, n = 5; MuSK-CAART+ agrin, n = 5; Wise-CAART, n = 2). **d**) U87-MG cells were stained with Calcein-AM before co-culture with T-cells. Viable cells (GFP^+^) were detected by fluorescence microscopy at 16 hours after co-culture with T-cells in the presence or absence of agrin. Representative images are shown from two individual experiments. **e**) Human IFNγ production was detected by ELISA in 16-hour co-culture supernatants from two individual experiments. One-way ANOVA with Holm-Sidak test for multiple comparisons: ***, p < 0.001.

Because in vivo biodistribution studies of human cellular immunotherapies are confounded by xGVHD and may not identify off-target interactions against human proteins, recombinant MuSK-Fc protein was used to screen an MPA comprising approximately 5,300 human membrane proteins overexpressed in mammalian cells, which identified binding to matrix metalloproteinase-16 (MMP16) in initial screening (Fig. 5c). Validation screens indicated overall low-level MuSK-Fc binding to MMP16 relative to positive and PD-1-Fc isotype controls (Fig. 5d,e), which was interpreted as negative (less than twofold higher than vector control) in one validation screen and positive (more than twofold higher than vector control) in a second validation screen. MuSK-CAART cytotoxicity was subsequently evaluated against U87-MG glioma cells, which express MMP16 as well as LRP4 (Extended Data Fig. 8a). As a positive control, we generated a CAAR comprising Wise, which interacts with LRP4 in an agrin-independent manner^29,30^, and verified Wise-CAART cytotoxicity against U87-MG cells (Extended Data Fig. 8b,c). In contrast to Wise-CAART, coincubation of MuSK-CAART with U87-MG cells ± agrin did not induce cell lysis or IFNγ production (Extended Data Fig. 8c-e). Additionally, screens of seven primary human or induced pluripotent stem cell (iPSC)-derived cells representing skin, vascular tissue, heart, brain/nerves, lung, liver and kidney, which were validated for MMP16 or LRP4 expression, did not identify specific cytolysis or IFNγ production (Fig. 5f-i).

**Fig. 6 ∣:**
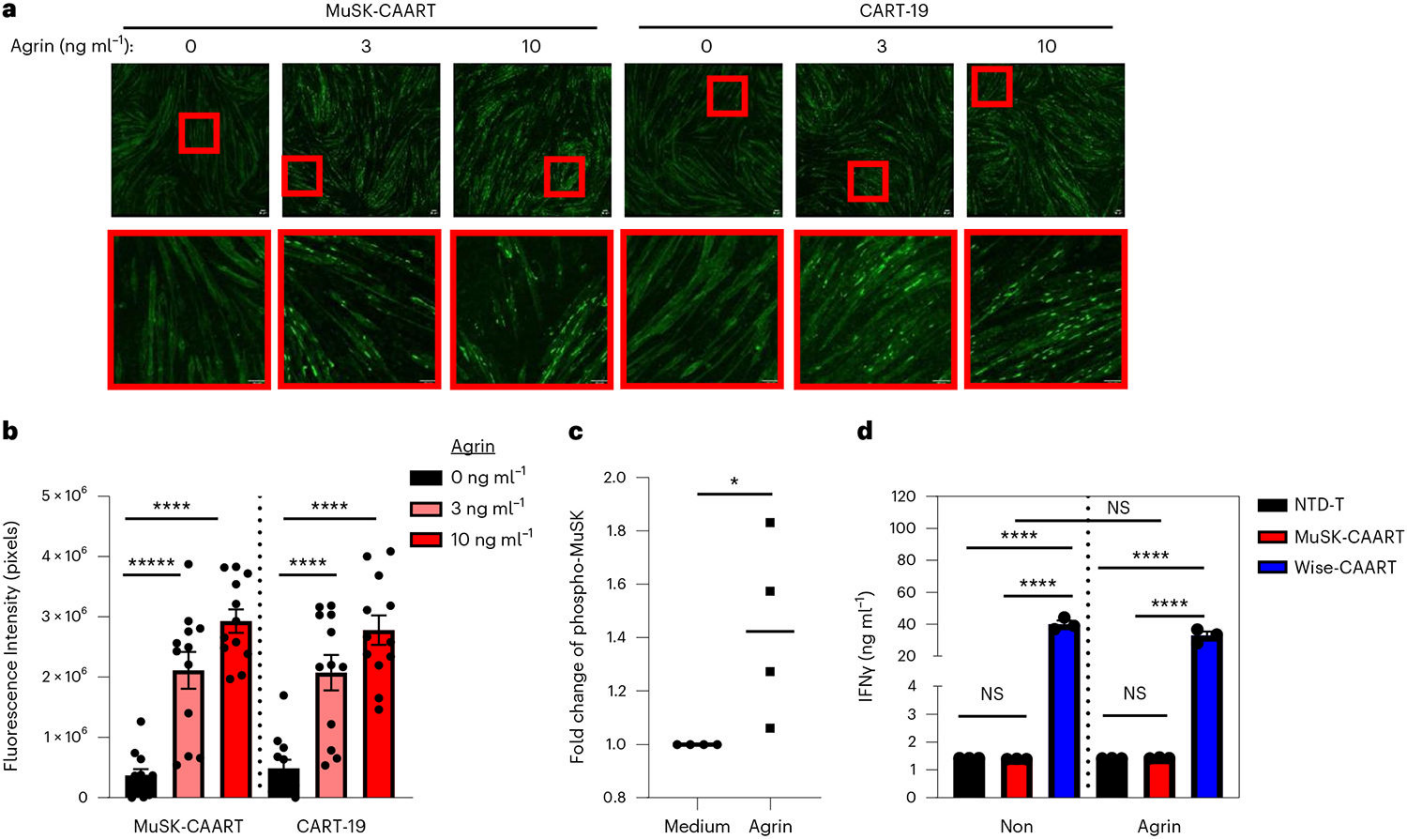
MuSK-CAART off-target effects on muscle are not observed. **a**, Effects of MuSK-CAART or CART-19 (E:T 10:1) on agrin-induced AChR clustering in C2C12 mouse myotubes were visualized by α-bungarotoxin staining (×25 magnification, top row, plus bottom inset (red boxes); scale bar, 50 μm). Representative images are shown from two individual experiments. **b**, AChR clustering was quantitated by fluorescence intensity, measured at six different sites in each image (error bars indicate mean ± s.e.m.). One-way ANOVA with the Holm–Sidak test for multiple comparisons. **c**, Differentiated primary human myotubes were incubated with agrin (5 ng ml^−1^) or medium alone. Agrin-induced MuSK phosphorylation was confirmed by phospho-MuSK ELISA. OD_450/570_ value relative to medium-alone control is shown. Mann–Whitney *U*-test (two-tailed). **d**, Human myotubes were coincubated with NTD-T, MuSK-CAART or Wise-CAART in the presence or absence of agrin for 24 hours. IFNγ production was measured in cell-culture supernatants by ELISA. Error bars indicate mean ± s.d. of triplicates. One-way ANOVA with the Holm–Sidak test for multiple comparisons. NS, *P* > 0.05; *P < 0.05; ****P < 0.0001.

To evaluate whether MuSK-CAART interferes with AChR clustering or causes muscle cell cytolysis due to potential *trans*-interaction with LRP4, mouse C2C12 myotubes were cocultured with MuSK-CAART or CART-19, which is known not to cause muscle cytotoxicity in humans. MuSK-CAART did not affect agrin-induced AChR clustering in C2C12 myotubes relative to CART-19 (Fig. 6a,b). Primary human muscle cells were also differentiated into myotubes, and MuSK activation by agrin was verified by MuSK phosphotyrosine enzyme-linked immunosorbent assay (ELISA) (Fig. 6c). IFNγ was not elevated in MuSK-CAART or NTD-T coculture supernatants, whereas a significant increase in human IFNγ was detected in Wise-CAART cocultures (Fig. 6d).

## Discussion

We report the development of a novel precision cellular immunotherapy for autoantigen-specific B cell depletion in MuSK MG. MuSK-CAART demonstrated specific cytotoxicity against B cells targeting each of the four MuSK domains (Fig. 1). By incorporating the complete MuSK ectodomain, MuSK-CAART is designed to eliminate autoimmune B cells targeting a broad range of MuSK epitopes.

A major difference in the application of MuSK-CAART and CART-19 to clinical practice is the presence of soluble autoantibodies that could have varying effects on MuSK-CAART function. Human anti-MuSK antibodies are predominantly IgG4, which are functionally monovalent and do not fix complement or activate antibody-dependent cellular cytotoxicity^17,31,32^. Data in Fig. 2a indicate that anti-MuSK antibodies modestly inhibit MuSK-CAART cytotoxicity in vitro, although cytotoxicity increases with longer coincubation times and higher effector to target ratios, while remaining specific. Cytotoxicity in the presence of soluble autoantibody was further confirmed in an EAMG model (discussed further below). Anti-MuSK IgG4 mAbs as well as IgG from patients with MuSK MG activate MuSK-CAART to produce IFNγ and/or proliferate (Fig. 2 and Extended Data Fig. 4). Anti-MuSK antibodies did not mediate indirect lysis of Fc-receptor-expressing cells by MuSK-CAART (Fig. 2e,f). Collectively, these data suggest that soluble autoantibodies could be beneficial by amplifying the infused MuSK-CAART dose (Figs. 2d and 3b and Extended Data Fig. 4c) and providing a survival signal in vivo; however, autoantibodies could also induce cytokine release syndrome (Fig. 2b,c and Extended Data Fig. 4b) and/or inhibit cytotoxicity (Fig. 2a and Extended Data Fig. 4a). In preclinical studies of desmoglein 3 (DSG3)-CAART for the treatment of mucosal pemphigus vulgaris, similar induction of CAART proliferation and IFNγ production by soluble autoantibodies occurred^21,33^, although data from the first four cohorts in the ongoing DSG3-CAART clinical trial (NCT04422912) indicate no cytokine release syndrome or dose-limiting toxicities, as well as a dose-related increase in DSG3-CAART persistence throughout the 28 days following infusion^34^, suggesting that soluble autoantibodies do not mediate adverse events or prevent DSG3-CAART engraftment. Nevertheless, to mitigate risk, dose escalation is planned in the MuSK-CAART phase 1 clinical study design.

To investigate the in vivo efficacy of MuSK-CAART, we used two complementary approaches. The NSG Nalm-6 xenograft model is a well-defined model that allows (1) evaluation of the cytolytic efficacy and engraftment of the human clinical product, MuSK-CAART, in comparison to clinically approved CART-19; (2) inclusion of anti-MuSK Nalm-6 cells that bind a broad range of epitopes relevant to MuSK MG and (3) sensitive real-time analysis of target cell burden by bioluminescence imaging. These studies indicate that MuSK-CAART demonstrates in vivo efficacy comparable to CART-19, including cytolysis of 13-3B5/anti-Ig1 Nalm-6 cells in the presence of matching soluble autoantibody that could inhibit MuSK-CAART cytotoxicity (Fig. 3). However, nearly 100% of Nalm-6 cells are MuSK-reactive, whereas MuSK-reactive B cells in patients with MuSK MG are rare (reported to be less than 0.15% of circulating IgG B cells^17^). Additionally, rare BCR-negative or BCR-low Nalm-6 cells, which persist despite enrichment for anti-MuSK BCR expression, proliferate and mediate delayed escape from MuSK-CAART in the NSG xenograft model (Extended Data Fig. 6). In humans, BCR-negative plasma cells would not be targeted by MuSK-CAART. However, MuSK autoantibody titers markedly decrease after therapy with rituximab, which also does not deplete plasma cells, suggesting that most anti-MuSK antibodies are produced by short-lived plasma cells that are continuously replenished from the CD20^+^ (and BCR^+^) memory B cell pool^11-13^. Additionally, studies have shown that BCR signaling and internalization are mutually exclusive and that anergic B cells downregulate their BCR by continuous recycling^35,36^, suggesting that either direct cytolysis of anti-MuSK B cells or selective pressure by MuSK-CAART to downregulate anti-MuSK BCRs may result in favorable therapeutic outcome.

To further evaluate MuSK-CAART in a preclinical autoimmune model, we expressed human MuSK-CAAR in murine T cells and compared its efficacy to anti-CD19 1D3-CART in an immunocompetent syngeneic MuSK EAMG model. Similar to human MuSK MG, anti-MuSK B cells comprised <0.2–1.5% of splenic IgG^+^ B cells and targeted all four MuSK ectodomains (Extended Data Fig. 7). Unlike human MuSK MG, both anti-MuSK IgG1 and IgG2c were induced in this model (Extended Data Fig. 7b). Murine IgG1 is functionally analogous to human IgG4 (ref. ^32^). Murine IgG2c fixes complement, which might mediate CAART destruction or cause toxicities that may not occur in patients with MuSK MG. Despite these limitations, MuSK-CAART specifically reduced anti-MuSK IgG but not total IgG or total B cells, indicating antigen-specific B cell depletion without previous lymphodepletion (Fig. 4). Anti-MuSK IgG reductions by MuSK-CAART and 1D3-CART were comparable, despite lower percentages of CD45.1^+^ T cells in MuSK-CAART versus 1D3-CART-treated mice (Fig. 4f). Differences in T cell engraftment were in part due to number of injected T cells in a subset of mice, but may also reflect IgG2c-mediated clearance of MuSK-CAART or the lower abundance of anti-MuSK versus CD19^+^ B cells, which induces less expansion of MuSK-CAART relative to 1D3-CART.

Multiple complementary approaches were used to screen for potential MuSK-CAART off-target effects (Figs. 5 and 6). Biodistribution studies in mice may identify unexpected organ cytotoxicity due to cross-reactivity with mouse proteins in their native context, but are limited by xGVHD and nonhomology with human proteins. MPAs allow high-throughput screening of thousands of human cell-surface proteins that may not otherwise be expressed in primary human cells or mice, but may identify irrelevant targets due to artifacts of protein overexpression. One of two MPA screens identified MuSK-Fc interaction with MMP16 (Fig. 5c-e), although follow-up screens against MMP16-expressing cells did not confirm MuSK-CAART cytotoxicity (Fig. 5f-i and Extended Data Fig. 8). These studies also indicated that MuSK, which physiologically interacts with LRP4 in *cis*, does not interact with LRP4 in *trans* on muscle and other primary cell types.

These data contributed to an investigational new drug application for MuSK-CAART as a novel precision cellular immunotherapy for the treatment of MuSK autoantibody-positive MG and informed a phase 1 clinical study design (NCT05451212). CAAR T cells may represent a platform technology that could be applied to numerous autoimmune and alloimmune B cell-mediated conditions.

## Methods

### Design and construction of plasmids

#### Lentiviral plasmids.

Lentiviral plasmid pTRPE (provided by the Penn Center for Cellular Immunotherapies) was modified to express MuSK-CAAR constructs and anti-MuSK BCRs as follows:

The human MuSK ectodomain (representing amino acids 24–495) was synthesized (Integrated DNA Technologies) with flanking 5′ BamHI and 3′ NheI restriction sites. Gene fragments were digested and purified using a PCR purification kit (Qiagen), then ligated into the pTRPE-DSG3-CAAR vector^33^ upstream of sequences encoding a glycine-serine linker, CD8α transmembrane, CD137 costimulatory and CD3ζ signaling domains. Wise (amino acids 24–206, UniProt Q6X4U4) was synthesized (Integrated DNA Technologies) and subcloned into pTRPE vector.

Anti-MuSK BCR 189-1 (ref. ^18^) (also known as MuSK 1A, anti-Ig2) and 13-3B5 (ref. ^17^) (anti-Ig1) was produced by synthesizing (Integrated DNA Technologies) the variable heavy and variable light chain sequences with flanking BamHI/NheI and XhoI/Bsu36I restriction sites. Gene fragments were digested, purified (PCR purification kit, Qiagen) and ligated into a pRRL4.IgG4 vector following previously published methods^33^, then subcloned into lentiviral plasmid pTRPE to generate pTRPE.IgG4.Lambda.189-1. Anti-MuSK BCRs 4A3 (ref. ^12^) (anti-Fz), 3-28 (refs. ^12,18^) (anti-Ig2) and 192-8 (human anti-Fz IgM, sequences provided by K.C.O.) were produced similarly, except that the kappa variable region was synthesized with flanking XhoI/BsiWI sites, and the kappa constant region was synthesized and cloned into a pGEM-T Easy vector (Promega) before ligation into pTRPE to generate pTRPE.IgG4.Kappa (4A3, 3-28 and 192-8). An anti-Ig3 mouse hybridoma (24C10) was produced by immunization of mice with the human MuSK ectodomain (Genscript) and the variable heavy and light chain genes sequenced (Genscript) and synthesized (Integrated DNA Technologies). Gene fragments were digested using BamHI/NheI (for the variable heavy chain) and XhoI/BsiWI (for the variable light chain), purified (PCR purification kit, Qiagen) and ligated into pTRPE.IgG4. Kappa vector.

Packaging plasmids pRSV-Rev and pGAG/POL, plus envelope plasmid Pcl VSVg (Nature Technology Corporation) were used with Lipofectamine 2000 (Life Technologies) for lentiviral preparation in 293T cells or Lenti-X 293T cells (Takara, 632180).

#### Antibody plasmids.

Variable heavy chain or variable light chain sequences of MuSK-specific antibodies were cloned into AbVec vectors (IgG4 heavy chain, kappa light chain or lambda light chain). A 1:1 mixture of variable heavy chain and variable light chain plasmids was cotransfected into 293T cells to produce recombinant monoclonal antibody. mAbs were purified from 293T culture supernatants using protein A chromatography (Invitrogen) according to the manufacturer’s recommendations.

#### Retroviral plasmids.

pMSGV1.1D3-28Z.1-3 mut was obtained from Addgene (107227)^28^. pMSGV1.MuSK-CAAR was generated by replacing the 1D3-28Z.1-3 mut insert with the MuSK-CAAR sequence. Then 30 μg of each plasmid was transfected into the Plat-E (Cell Biolabs) packaging cell line to produce retroviruses.

### Patient samples

#### Patient characteristics.

MG3, a 57-year-old female, chronic active disease. MG5, a 34-year-old female, chronic active disease. Venipuncture was performed under a protocol approved by the University of Pennsylvania Institutional Review Board.

#### MuSK MG IgG purification.

IgG was purified from plasma from patients MG3 and MG5 by protein A chromatography (Invitrogen) according to the manufacturer’s recommendations.

### Evaluation of MuSK monoclonal antibody and IgG titers from patients with MG

Relative titers of each recombinant anti-MuSK monoclonal antibody or purified plasma IgG from patients with MG (MG3 and MG5) was evaluated using a Luminex-based assay. In brief, purified MG3 IgG (0.85 mg ml^−1^) and MG5 IgG (2.2 mg ml^−1^) were diluted 1:10, 1:50 and 1:100. Recombinant anti-MuSK monoclonal antibodies were evaluated at 0.1, 0.2, 0.5 and 1.5 μg ml^−1^ concentrations. Diluted samples were added to MuSK ectodomain (aa 24–495)-coupled microspheres and incubated for 1 h at room temperature. After washing samples, 5 μg ml^−1^ antihuman IgG-Biotin was added and incubated for 1 h at room temperature, followed by incubation with 100 μl of streptavidin-PE (4 μg ml^−1^) for 45 min. After washing, beads were resuspended in 100 μl of washing buffer and 60 μl was analyzed using a Luminex 100TM/200TM analyzer according to the manufacturer’s recommendations.

### In vitro transduction and expansion of CAR/CAAR T cells

#### In vitro transduction and expansion of human CAR/CAAR T cells.

Bulk (mixed CD4/CD8) primary human T cells (from Penn Human Immunology Core or leukapheresis (Stem Express)) were cultured in human T cell culture media supplemented with 100 IU ml^−1^ rhIL-2 (Proleukin) (CTS OpTmizer media (Invitrogen, A1048501) plus 5% human AB serum (Gemini Bio-Products, 100–512) or Roswell Park Memorial Institute (RPMI) media supplemented with 10% FBS, 10 mM HEPES, 1% penicillin/streptomycin and 1% GlutaMax). T cells were activated/selected with anti-CD3/CD28-coated paramagnetic beads (Thermo Fisher Scientific, 40203D) at a 3/1 bead/cell ratio. Lentivirus was added at 24 h after activation, and cells were expanded in either static culture or a Xuri Bioreactor (GE Healthcare Lifesciences) until day 9 to day 10 after activation, with media changes approximately every 2 days and magnetic bead removal before cryostorage. Expression of anti-CD19 CAR or MuSK-CAAR on human T cells was detected using CD19-Biotin with Streptavidin-PE, CD19-PE or recombinant anti-MuSK antibodies (189-1, 24C10 or 4A3) with antihuman IgG4-APC or antimouse IgG1-PE.

#### In vitro transduction and expansion of mouse CAR/CAAR T cells.

Mouse T cells (CD45.1^+^ C57BL6/J, Jackson Laboratory, strain 002014) were purified using a CD3 T cell enrichment kit (R&D Systems) and cultured using mouse T cell culture media (RPMI-10 media supplemented with 10% FBS, 10 mM HEPES, 1% penicillin/streptomycin, and 1% GlutaMAX). T cells (10^6^ cells per ml) were activated for 36 h with Dynabead Mouse T-Activator CD3/CD28 (Thermo Fisher Scientific, 11452D) in media supplemented with 50 IU ml^−1^ rhIL-2 and 10 ng ml^−1^ rhIL-7 (BioLegend, 581902), plus fresh 20 μM 2-mercaptoethanol. Polystyrene nontreated plates were coated with 30 μg ml^−1^ RetroNectin (Takara) at 4 °C overnight then blocked with mouse T cell culture media for 30 min at room temperature, followed by centrifugation with retroviral supernatant by centrifugation at 3,000 *g* for 2 h at 4 °C. Transduction was conducted on days 1 and 2 after activation by adding T cells directly onto retrovirus-coated wells. Plates were centrifuged at 300 *g* for 10 min at room temperature, then placed in a cell-culture incubator overnight (first round transduction). Second round transduction was performed similarly, except T cells were incubated 4–6 h after second round transduction in mouse T cell culture media supplemented with 10 ng ml^−1^ rhIL-7 and 10 ng ml^−1^ rhIL-15 (BioLegend, 570302) for an additional 3 days. Media was replaced the next day and as needed to maintain the cell concentration between 1 and 2 × 10^6^ cells per ml. Expression of anti-CD19 CAR or MuSK-CAAR on mouse T cells was detected on day 4 after activation using antimouse IgG(H+L)-APC (Jackson ImmunoResearch) or APC-conjugated recombinant anti-MuSK antibody (189-1). Mouse T cells were injected on day 5 after activation.

### Pharmacologic and toxicologic effects of soluble anti-MuSK antibodies

#### MuSK-CAART cytotoxicity in the presence of MuSK MG IgG.

Donor-matched MuSK-CAART and NTD-T were incubated with BCR-negative Nalm-6 control cells or mixture of Nalm-6 MuSK target cells (1:1:1:1 ratio of Nalm-6 13-3B5, Nalm-6 3-28, Nalm-6 24C10 and Nalm-6 192-8) at E:T ratio of either 1:1 or 10:1 for 24 h. Purified IgG from two patients with MuSK MG (MG3 and MG5) was added at a final concentration of 10 mg ml^−1^ IgG before coincubation. MuSK-CAART cytotoxicity was evaluated at 8 and 24 h using luciferase-based killing assay. Coculture supernatants were harvested after completing the final plate reading at 24 h and stored at −20 °C for IFNγ ELISA.

#### IFNγ production and proliferation of MuSK-CAART by soluble antibodies.

Soluble anti-MuSK antibody-induced MuSK-CAART activation and proliferation was evaluated by IFNγ ELISA or Cell Trace Violet (CTV) cellular labeling, respectively. For IFNγ ELISA, donor-matched MuSK-CAART and NTD-T were incubated with a mixture of recombinant anti-MuSK monoclonal antibodies (1:1:1:1 of 13-3B5, 3-28, 24C10 and 192-8) for 24 h. For the CTV cellular labeling, T cells were labeled with CTV Cell Proliferation Kit (Invitrogen) and activated with a mixture of anti-MuSK monoclonal antibodies for 96 h before analysis by flow cytometry.

#### IncuCyte assay.

Monocytes and NK cells mixed with green caspase-3/7 dye were incubated in media containing relevant IgGs, as well as effector cells. The normal human IgG amount (negative control) and UCHT1 (a positive control, anti-CD3 antibody) was matched to the equivalent total amount of mixed anti-MuSK monoclonal antibodies or purified plasma IgG from patients with MG. Monocytes and NK cells were cocultured with MuSK-CAART or NTD-T at a 5:1 E:T ratio. Anti-MuSK monoclonal antibodies 13-3B5, 3-28, 192-8 were mixed at 1:1:1 ratio. Cocultures were monitored for 48 h using an IncuCyte S3 system (Sartorius) and images were taken every 2 h with a ×20 objective, then dead cells (green positive) were counted at each timepoint from four fields of images per each well. The number of caspase-positive cells is equal to the mean number of green cell counts in each imaging field.

### Luciferase-based in vitro cytotoxicity assay

#### Luciferase-based killing assay.

Click-beetle green luciferase expressing cells or luciferase^+^ U87-MG cells (ATCC, HTB14Luc2) were cocultured with engineered T cells or donor-matched NTD-T at an indicated effector:target (E:T) ratio. At 3 h after coculture, luciferase substrate (d-luciferin potassium salt, GoldBio) was directly added to each well and emitted light was measured on a luminescence plate reader (BioTek, Synergy HTX microplate reader) at indicated timepoints. The percentage of specific lysis was calculated using the luciferase activity of 5% SDS-treated cells as maximum cell death and media alone as spontaneous cell death using the formula: specific lysis (%) = 100 × ((experimental data – maximum death data)/(maximum death data – spontaneous death data)).

### Ethics statement for animal research

All studies involving animals were performed under a protocol approved by the University of Pennsylvania Institutional Animal Care and Use Committee.

### In vivo MuSK-CAART evaluation using NSG Nalm-6 xenograft models

#### Target cell and T cell injection.

NSG (NOD.Cg-*Prkdc^scid^IL2rg^tm1Wjl^*/SzJ) mice received 600 mg kg^−1^ i.v. immunoglobulin (Privigen, IVIg) via tail-veil injection on day −2 and day −1 before target cell injection to prevent Fc-mediated Nalm-6 clearance. On day 0, three cohorts of NSG mice each received 10^6^ Nalm-6 target cell line(s) via tail-vein injection as follows: (1) Nalm-6 mixed (a 1:1:1:1 mixture of Nalm-6 13-3B5, Nalm-6 3-28, Nalm-6 24C10 and Nalm-6 192-8 or Nalm-6 4A3 cells, (2) Nalm-13-3B5 and (3) Nalm-6 13-3B5*. Nalm-6 13-3B5* cells were generated by introducing 13-3B5 IgG4 heavy chain (without a membrane anchor) into Nalm-6 13-3B5 expressing 13-3B5 IgG4 heavy chain (membrane-bound form) and 13-3B5 light chain, resulting in 13-3B5 IgG4 antibody-secreting cells that retained cell-surface 13-3B5 BCR expression (Extended Data Fig. 5). Donor-matched frozen human T cells (NTD-T, CART-19 and MuSK-CAART) were thawed 1 day before the treatment in human T cell culture media supplemented with 100 IU of rhIL-2. On day 4 after target cell injection, 10^7^ human T cells were injected via tail vein. Two different infusion products from the same donor were used in mixed (Fig. 3a) or 13-3B5/13-3B5* (Fig. 3b) Nalm-6 experiments. IVIg was injected every 2–3 days in mixed and 13-3B5 Nalm-6-engrafted mice.

#### Bioluminescence imaging.

Bioluminescence was measured with a Xenogen IVIS Lumina S3 (Caliper Life Sciences) from day 1 after target cell injection and every 2–3 days thereafter by injecting d-Luciferin potassium salt (Gold Bio) intraperitoneally at a dose of 150 mg kg^−1^. Mice were anesthetized with 2% isoflurane and luminescence was serially measured at 1 min intervals for 7 min or until signals start to decrease in an automatic exposure mode. Total flux in the peak image was quantified using Living Image 4.4 (PerkinElmer) by drawing rectangles from head to 50% of the tail length. Radiance unit of p s^−1^ cm^2^ sr^−1^ = number of photons per second per square centimeter that radiate into a solid angle of one steradian.

#### Human anti-MuSK antibody ELISA.

Serum samples in NSG mice with Nalm-6 13-3B5* were collected at day 5 and day 15 after target cell injection in K_2_EDTA tubes for MuSK antibody ELISA. To detect human anti-MuSK IgG4, the histidine-tagged recombinant extracellular domain of human MuSK (aa 24–495, R&D Systems, catalog no. 10189-MK) was coated on ELISA plates in PBS overnight at 4 °C at a concentration of 5 μg ml^−1^. Plates were washed with washing buffer (Invitrogen, catalog no. 00-0400-59), and blocked with Pierce Protein-Free (PBS) Blocking Buffer (Thermo Scientific, catalog no. 37572). Mouse serum samples were evaluated at a dilution of 1:50 to 1:100 in comparison to a 13-3B5 purified recombinant human monoclonal IgG4 antibody as a reference standard for quantitation. Antihuman IgG (H+L) HRP (Bethyl, catalog no. A80-119P) was used to detect human antibodies. Plates were protected from the light and placed in the dark for 2 h. After washing plates three times, 100 μl of TMB (Thermo Scientific, 34028) was added for 30 min. Plate reading were conducted using ELISA reader (Tecan, Infinite F-50) within 15 min after adding stop solution (Invitrogen, SS04).

#### Flow cytometry analysis.

Lymphocytes were isolated from cranial bone marrow using a previously reported protocol^37^. In brief, the calvaria was cut into small pieces using sterile scissors and dissociated in PBS + 2% FBS with a pestle. Spleens were harvested from mice, washed in PBS and cut into 0.5 mm cubes in ice-cold PBS. Spleen or bone marrow isolates were transferred to a 70 μm cell strainer (Falcon, catalog no. 352350); cells were washed with PBS and resuspended in red blood cell lysis buffer (BioLegend, catalog no. 420301). Cells were stained for 30 min on ice using the following antibodies: anti-CD3 BV711 or anti-CD3-AF647 (clone okt3, BD Biosciences, catalog nos. 750983 and 566686)), anti-MuSK PE (clone 189-1 or 24C10), antihuman Ig light chain λ PE (clone MHL-38, BioLegend, catalog no. 316608), antihuman Ig light chain κ APC (clone MHK-49, BioLegend, catalog no. 316510), antihuman IgG PE (BD Biosciences, catalog no. 555787) and/or antimouse IgG APC (clone A85-1, BD Biosciences).

#### Direct immunofluorescence.

The diaphragm was collected at the time of tissue harvest and embedded in optimal cutting temperature medium (Tissue-Tek); tissue blocks were frozen on dry ice before storage at −80 °C. Next, 9 μm sections were cut onto Superfrost/Plus glass slides (Fisher Scientific) and stored at −80 °C. Before staining, slides were equilibrated to room temperature, washed twice in PBS (Gibco) and blocked in PBS containing 2% BSA. Slides were stained using FITC-conjugated antihuman IgG (BioLegend) at a dilution of 1:200 in blocking solution and washed with PBS. Binding of IgG was visualized with a Keyence imaging system (BZ-X710) and software (BZ-X Viewer).

### Syngeneic MuSK EAMG model

#### Immunization and boosting schedule.

CD45.2^+^ C57BL/6J mice (Jackson Laboratory, strain 000664) were immunized with MuSK Ig1–Ig2 ectodomain fragment on day 0 and boosted with MuSK Ig1-Fz full-length ectodomain protein on day 26. In initial experiments, 20 mg kg^−1^ busulfan was injected on day 34 in a subset of mice to evaluate effects on induced antibody titer and MuSK-CAART activity and engraftment. On day 35, 8 × 10^6^ MuSK-CAART, 16 × 10^6^ anti-CD19-CART (1D3) or 8 × 10^6^ NTD-T in set 1 or 20 × 10^6^ cells each in set 2 were administered via i.v. injection. Analyses in Fig. 4a,b,f were performed 2 or 4 weeks after T cell treatment (set 2 or 1, respectively).

#### ELISA for total and subclass-specific mouse anti-MuSK IgG and total mouse IgG.

Blood samples were collected weekly. To detect mouse anti-MuSK antibodies in the syngeneic MuSK EAMG model, mouse plasma (diluted 1:100 in PBS) was incubated on MuSK protein-coated plates and subsequently detected with antimouse IgG-HRP (diluted 1:5,000, abcam, ab7061). Mouse anti-MuSK monoclonal antibody (clone 4A3) was used as a reference standard control across experiments. Goat antimouse IgG1, IgG2b, IgG2c or IgG3-HRP (SouthernBiotech) was used as a secondary antibody reagent to determine anti-MuSK IgG subclasses. Total mouse IgG was measured by ELISA following manufacturer’s protocols (Invitrogen, CAT), after diluting sera 1:10,000 in PBS.

#### ELISpot.

The frequency of anti-MuSK B cells in the spleen of MuSK-immunized mice were conducted using Mouse IgG ELISpot-basic kit (Mabtech, 3825-2H) according to the manufacturer’s protocol. Briefly, splenocytes were prestimulated with a mixture of R848 (1 μg ml^−1^) and rmIL-2 (10 ng ml^−1^) for 48 h. After prestimulation, cells were washed and resuspended in medium, then 10,000 or 100,000 B cells were plated in ELISpot wells precoated either with anti-IgG antibodies (total IgG B cells) or MuSK protein (10 μg ml^−1^), respectively.

#### Flow cytometry analysis.

Cells were isolated from the spleen and lymph nodes after red blood cell lysis and stained with anti-CD45.1-FITC (BioLegend, 110706), anti-CD45.2-PECy7 (BioLegend, 109830), anti-CD3ε-BV421 (BioLegend, 100227) and anti-CD19-APC (BioLegend, 115512) for 30 min on ice.

### Evaluation for off-target interactions of MuSK-CAART

#### Calcein-AM staining.

U87-MG cells, cultured to 70–90% confluency in a 12-well plate, were stained with 0.1–1 μM of Calcein-AM following the manufacturer’s protocol (BD Pharmingen), then coincubated for 20–24 h with 1 × 10^6^ MuSK-CAART, Wise-CAART or NTD-T. Then 5 nM of neuronal agrin was added to U87-MG culture supernatants at least 30 min before coculture.

#### Off-target toxicity against differentiated muscle cells.

Primary human skeletal muscle cells (ZenBio, SKB-F) were differentiated for 6–7 days using skeletal muscle cell differentiation medium (ZenBio, SKM-D). Next, 5 nM of neural agrin (R&D Systems, catalog no. 550-AG/CF) was added into differentiated primary human skeletal muscle cells for 16 h. MuSK phosphorylation was detected by ELISA (RayBiotech, catalog no. PEL-MUSK-Y-1) according to the manufacturer’s protocol. Optical density (OD_450/570_) values were normalized by total protein concentration in each sample. Donor-matched NTD-T, MuSK-CAART and Wise-CAART were added and cocultured for 22–26 h. For some experiments, neural agrin was added at least 30 min before T cell coculture.

Evaluation of AChR clustering in C2C12 myotubes was performed by Invivotek, LLC. C2C12 mouse myoblast cells (ATCC 70024392) were maintained in DMEM supplemented with 20% FBS and 1% penicillin/streptomycin. To induce myotube differentiation, C2C12 cells (80–90% confluent) were cultured in DMEM plus 2% horse serum and 0.5% penicillin/streptomycin. Agrin-induced AChR clustering was examined by incubating C2C12 myotubes in 1:1 DMEM differentiation media and RESGRO serum-free culture medium (EMD Millipore, SCM002) supplemented with varying concentrations of agrin (R&D Systems, catalog no. 550-AG/CF) for 14 h at 37 °C. AChR staining was performed using 1 μg ml^−1^ AlexaFluor 488-labeled α-bungarotoxin (Invitrogen catalog no. B13422). Myotubes were fixed with 4% paraformaldehyde for 20 min at room temperature and imaged under mounting medium (Vector Laboratories) using a Leica TCS SP8 multiphoton confocal microscope. Fluorescence was quantified (Fiji-Image J) as corrected total cell fluorescence = integrated density – (area of selected cell × mean fluorescence of background).

#### Off-target toxicity against primary human cells.

MuSK-CAART reactivity against two different donor batches of primary or iPSC-derived human cells was performed by Charles River Discovery Research Services, including iPSC-derived iCell cardiomyocytes (Fuji Cellular Dynamics no. R1007/R1106), iPSC-derived iCell GABANeurons (Fuji Cellular Dynamics, no. R1013/R1011), human bronchial epithelial cells (HBEC, Lonza, no. CC-2540), primary hepatocytes (InnoProt, no. P10651), human renal epithelial cells (HREC, InnoProt, no. P10664), normal human epidermal melanocytes (NHEM, Lonza no. CC-2586 and LifeLine no. FC-0030) and human umbilical vascular endothelial cells (HUVEC, Lonza, no. CC-2517). BCR^−^ Nalm-6 cells and Nalm-6 3-28 cells were used as a negative control and a positive control, respectively. Primary or iPSC-derived human cells were cocultured for 24 h with two sets of donor-matched NTD-T and MuSK-CAART at an E:T ratio of 5:1.

MuSK-CAART cytotoxicity was detected at 24 h using either an HCA with Nalm-6 cells, cardiomyocytes, hepatocytes and HBEC or flow cytometry analysis with Nalm-6 cells, HREC, HUVEC and NHEM, respectively. 1 μM staurosporin or 10 μM bortezomib (for HREC) was used as a toxin control. For HCA, CAR/CAAR T cells were stained with CellTracker Deep Red dye for 24 h, followed by incubation with 10 μg ml^−1^ Hoechst and 4 μg ml^−1^ propidium iodide in PBS supplemented with 0.5% BSA for 10 min at room temperature. Cells were imaged using the GE Healthcare IN Cell Analyzer 6000 (×10 magnification). Brightfield, ultraviolet, dsRed and Cy5 channels were used to image brightfield, Hoechst staining, propidium iodide staining and CEllTracker staining, respectively. Cell type-specific HCA was used to quantify live target cells (IN Cell Developer Toolbox software (v.1.9.1)). Nuclear area of nonviable target cells was determined based on propidium iodide staining and subtracted from total target cell nuclear area. For flow cytometry analysis, target cells were stained with 1 μM CellTracker Deep Red dye then incubated with 0.5 μg ml^−1^ propidium iodide in the presence of precision count beads before flow cytometry (Agilent NovoCyte Quanteon, FlowJo software v.10). Absolute count of live target cells was normalized to absolute bead count.

#### Measurement of IFNγ.

Secreted IFNγ in coculture supernatants was detected by ELISA (BioLegend) or with a Luminex bead array platform (Thermo Fisher) according to the manufacturer’s instructions. All samples were analyzed in triplicate and compared against multiple internal standards, with a seven-point standard curve.

#### MPA screen.

MPA was performed by Integral Molecular. In brief, a flow cytometry assay was used to assess the binding targets of MuSK extracellular domains (amino acids 24–495) linked with human Fc (MuSK-Fc Chimera) among 5,300 human membrane proteins overexpressed in 293T cells (permeabilized before MuSK-Fc incubation and flow cytometry detection). To validate potential off-targets of MuSK-Fc Chimera, human embryonic kidney 293T cells were transfected with plasmids expressing the respective targets, vector alone (negative control), PD-1-Fc isotype control or membrane-bound protein A construct and anti-MuSK BCR (4A3 clone) (positive controls). After confirming target protein expression, titration assays were conducted to validate a potential off-target of MuSK-Fc Chimera at different concentrations.

#### Biodistribution assay.

A GLP-compliant biodistribution study to evaluate the safety of MuSK-CAART in NSG mice was performed by Pharmaseed Ltd (Ness Ziona, Israel). On days −4 and −3, mice were pretreated with i.v. administration of i.v. immunoglobulin (IVIg). Target cells (1 × 10^6^ Nalm-6 3-28 cells) were administered i.v. on day −2. After target cell engraftment, IVIg was administered i.p. every 2–3 days up to day 18. Two days after target cell administration, assigned day 1, the mice were injected i.v. with either vehicle (*n* = 4), 3 × 10^6^ or 1 × 10^7^ MuSK-CAART (*n* = 24 for each dose), 1 × 10^7^ CART-19 (*n* = 24) or 1 × 10^7^ NTD-T (*n* = 8) at a dose volume of 200 μl per mouse. Male and female mice were distributed equally. Effector cell (NTD-T, MuSK-CAART or CART-19) administration was performed on day 1 and mouse harvest for pathologic evaluation, serum chemistry and complete blood count was performed on days 15, 36 and at study termination on day 61. Weights and clinical observations were performed biweekly. Nalm-6 distribution was detected using bioluminescence imaging. Organs were fixed in formalin for hematoxylin and eosin staining and for pathologist evaluation (Pharmaseed). Histopathological changes of MuSK-CAART and control mice were described and scored using semiquantitative grading of five grades (0–4): grade 0, normal; grade 1, minimal; grade 2, mild; grade 3, moderate and grade 4, severe.

## Extended Data

## Data Availability

The data that support the findings of this study are available from the corresponding author upon reasonable request. Source data are provided with this paper.
