# Author Correction: Precision targeting of autoantigen-specific B cells in muscle-specific tyrosine kinase myasthenia gravis with chimeric autoantibody receptor T cells

**DOI:** 10.1038/s41587-024-02502-x

**Published:** 2024-11-14

**Authors:** Sangwook Oh, Xuming Mao, Silvio Manfredo-Vieira, Jinmin Lee, Darshil Patel, Eun Jung Choi, Andrea Alvarado, Ebony Cottman-Thomas, Damian Maseda, Patricia Y. Tsao, Christoph T. Ellebrecht, Sami L. Khella, David P. Richman, Kevin C. O’Connor, Uri Herzberg, Gwendolyn K. Binder, Michael C. Milone, Samik Basu, Aimee S. Payne

**Affiliations:** 1 Department of Dermatology, Perelman School of Medicine, University of Pennsylvania, Philadelphia, PA USA; 2 Cabaletta Bio, Philadelphia, PA USA; 3 Department of Neurology, Perelman School of Medicine, University of Pennsylvania, Philadelphia, PA USA; 4 Department of Neurology, University of California – Davis, Davis, CA USA; 5 Departments of Neurology and Immunobiology, Yale School of Medicine, New Haven, CT USA; 6 Department of Pathology and Laboratory Medicine, Perelman School of Medicine, University of Pennsylvania, Philadelphia, PA USA

**Keywords:** Drug development, Immunotherapy, Autoimmunity, Regenerative medicine

Correction to: *Nature Biotechnology* 10.1038/s41587-022-01637-z, published online 19 January 2023.

In the version of this article initially published, the Acknowledgements did not include thanks for research support from the Myasthenia Gravis Foundation of America Nancy Law Impact Award as has now been amended in the HTML and PDF versions of the article.

