# Supplementary material for: Precision targeting of autoantigen-specific B cells in muscle-specific tyrosine kinase myasthenia gravis with chimeric autoantibody receptor T cells
Source: Nat Biotechnol. 2023 Jan 19;41(9):1229–38. doi: 10.1038/s41587-022-01637-z (PMC10354218; doi:10.1038/s41587-022-01637-z)
Supplement: Supplementary file 1 — Reporting Summary [file 41587_2022_1637_MOESM1_ESM.pdf]

nature research

Corresponding author(s): Aimee S. Payne

Last updated by author(s): Dec 1, 2022

Reporting Summary

Nature Research wishes to improve the reproducibility of the work that we publish. This form provides structure for consistency and transparency in reporting. For further information on Nature Research policies, see our Editorial Policies and the Editorial Policy Checklist.

Statistics

For all statistical analyses, confirm that the following items are present in the figure legend, table legend, main text, or Methods section.

n/a	Confirmed	
☐	☒	The exact sample size (*n*) for each experimental group/condition, given as a discrete number and unit of measurement
☐	☒	A statement on whether measurements were taken from distinct samples or whether the same sample was measured repeatedly
☐	☒	The statistical test(s) used AND whether they are one- or two-sided<br>*Only common tests should be described solely by name; describe more complex techniques in the Methods section.*
☒	☐	A description of all covariates tested
☐	☒	A description of any assumptions or corrections, such as tests of normality and adjustment for multiple comparisons
☐	☒	A full description of the statistical parameters including central tendency (e.g. means) or other basic estimates (e.g. regression coefficient) AND variation (e.g. standard deviation) or associated estimates of uncertainty (e.g. confidence intervals)
☐	☒	For null hypothesis testing, the test statistic (e.g. *F*, *t*, *r*) with confidence intervals, effect sizes, degrees of freedom and *P* value noted<br>*Give P values as exact values whenever suitable.*
☒	☐	For Bayesian analysis, information on the choice of priors and Markov chain Monte Carlo settings
☒	☐	For hierarchical and complex designs, identification of the appropriate level for tests and full reporting of outcomes
☒	☐	Estimates of effect sizes (e.g. Cohen's *d*, Pearson's *r*), indicating how they were calculated

Our web collection on statistics for biologists contains articles on many of the points above.

Software and code

Policy information about availability of computer code

Data collection	Flow cytometry data were acquired on a LSR II flow cytometer (BD Bioscience).
Data analysis	Statistical analyses were performed using GraphPad Prism (v9.3.0). Flow cytometry data were analyzed using Flowjo (V10.8.1, Tree Star)

For manuscripts utilizing custom algorithms or software that are central to the research but not yet described in published literature, software must be made available to editors and reviewers. We strongly encourage code deposition in a community repository (e.g. GitHub). See the Nature Research guidelines for submitting code & software for further information.

Data

Policy information about availability of data

All manuscripts must include a data availability statement. This statement should provide the following information, where applicable:

- Accession codes, unique identifiers, or web links for publicly available datasets
- A list of figures that have associated raw data
- A description of any restrictions on data availability

The authors confirm that the data supporting the findings of this study are available within the article and its supplementary materials

Field-specific reporting

Please select the one below that is the best fit for your research. If you are not sure, read the appropriate sections before making your selection.

☒ Life sciences ☐ Behavioural & social sciences ☐ Ecological, evolutionary & environmental sciences

For a reference copy of the document with all sections, see nature.com/documents/nr-reporting-summary-flat.pdf

Life sciences study design

All studies must disclose on these points even when the disclosure is negative.

Sample size	Sample sizes were not predetermined based on statistical methods, but were chosen according to the standards of the field (at least three independent replicates).
Data exclusions	Data exclusions are indicated in legends for Figure 3c-d, Figure 4d-e (reflecting exclusion of mice that were sacrificed at early timepoints for interim analysis and one mouse with low venipuncture volume precluding analysis.)
Replication	Results were replicated across multiple experiments as indicated in Figure legends.
Randomization	For the Nalm-6 NSG mouse model, treatment groups were randomly selected by cage. For the syngeneic mouse model, treatment groups were selected such that groups had similar autoantibody titers at the time of the treatment.
Blinding	The experiments were not blinded. Experimental design for full blinding or partial blinding is not feasible in this study.

Reporting for specific materials, systems and methods

We require information from authors about some types of materials, experimental systems and methods used in many studies. Here, indicate whether each material, system or method listed is relevant to your study. If you are not sure if a list item applies to your research, read the appropriate section before selecting a response.

Materials & experimental systems

n/a	Involved in the study	
☐	☒	Antibodies
☐	☒	Eukaryotic cell lines
☒	☐	Palaeontology and archaeology
☐	☒	Animals and other organisms
☐	☒	Human research participants
☒	☐	Clinical data
☒	☐	Dual use research of concern

Methods

n/a	Involved in the study	
☒	☐	ChIP-seq
☐	☒	Flow cytometry
☒	☐	MRI-based neuroimaging

Antibodies

Antibodies used

1. Mouse anti-MuSK antibody (clone: 4A3, home-made): flow cytometer (FC) 1:1000
2. Human anti-MuSK antibodies (clones: 13-3B5, 189-1, 3-28, 24C10, 192-8, and 4A3, home-made)
3. Goat anti-human IgG(H+L) HRP (Bethyl laboratories, Cat#A80-119P)
4. Mouse anti-human CD3 BV711 (Clone:okt3, BD Biosciences, Cat#750983)
5. Mouse anti-human CD3 AF647 (Clone:okt3, BD Biosciences, Cat#566686),
6. Mouse anti-human Ig light chain lambda-PE (Clone: MHL-38, BioLegend, Cat#316608);
7. Mouse anti-human Ig light chain kappa-APC (Clone: MHK-38, BioLegend, Cat#316510),
8. Mouse anti-human IgG-PE (BD Biosciences, Cat#555787);
9. Rat anti-mouse IgG1-APC (Clone A85-1, BD Biosciences, Cat#560089);
10. Mouse anti-mouse CD45.1-FITC (clone: A20, BioLegend, Cat#110706);
11. Mouse anti-mouse CD45.2-PECy7 (clone: 104, BioLegend, Cat#109830);
12. Rat anti-mouse CD3e-BV421 (clone: 17A2, BioLegend, Cat#100227);
13. Rat anti-mouse CD19-APC (clone: 6D5, BioLegend, Cat#115512);
14. AlexaFluor 488-labeled alpha-bungarotoxin (Invitrogen, Cat#B13422);
15. Donkey anti-mouse IgG-HRP (abcam, Cat#ab7061);
16. Goat anti-Mouse IgG1 (Cat#1071-05), IgG2b (Cat#1090-05), IgG2c (Cat#1078-05), and IgG3 (Cat#1101-05) from SouthernBiotech;
17. Mouse anti-human IgG-PE (BD Biosciences, Cat#555787);
18. Mouse anti-human IgG4 Fc-AF647 (SouthernBiotech, Cat#9200-31);
19. PE-labeled Human CD19 (20-291) protein (ACROBiosystems, Cat#CD9-HP2H3).

Validation

1. Mouse anti-MuSK antibody (clone: 4A3, home-made): use of antibody for FC (1:1000 dilution) was validated in MuSK-overexpressing 293T cell line in Takara et al., 2019. JCI Insight. 4(12): e127167

2. Human anti-MuSK antibodies (clones: 13-3B5, 189-1, 3-28, 24C10, 192-8, and 4A3, home-made): use of antibodies for FC (1:1000 dilutions except 189-1 (1:2000 dilution)) was validated in MuSK-CAAR overexpressing Jurkat cells in this manuscript.
3. Goat anti-human IgG(H+L) HRP (Bethyl laboratories, Cat#A80-119P): use of antibody for ELISA (1:10,000-1:100,000 dilution) was validated by Bethyl laboratory.
4. Mouse anti-human CD3 BV711 (Clone:okt3, BD Biosciences, Cat#750983): use of antibody for FC (1:1000 dilution) was validated by BD Biosciences.
5. Mouse anti-human CD3 AF647 (Clone:okt3, BD Biosciences, Cat#566686): use of antibody for FC (1:1000 dilution) was validated by BD Biosciences.
6. Mouse anti-human Ig light chain lambda-PE (Clone: MHL-38, BioLegend, Cat#316608): use of antibody for FC (1:1000 dilution) was validated by BioLegend.
7. Mouse anti-human Ig light chain kappa-APC (Clone: MHK-38, BioLegend, Cat#316510): use of antibody for FC (1:1000 dilution) was validated by BioLegend.
8. Mouse anti-human IgG-PE (BD Biosciences, Cat#555787): use of antibody for FC (1:1000 dilution) was validated by BD Biosciences.
9. Rat anti-mouse IgG1-APC (Clone A85-1, BD Biosciences, Cat#560089): use of antibody for FC (1:1000 dilution) was validated by BD Biosciences.
10. Mouse anti-mouse CD45.1-FITC (clone: A20, BioLegend, Cat#110706): use of antibody for FC (1:1000 dilution) was validated by BioLegend.
11. Mouse anti-mouse CD45.2-PECy7 (clone: 104, BioLegend, Cat#109830): use of antibody for FC (1:1000 dilution) was validated by BioLegend.
12. Rat anti-mouse CD3e-BV421 (clone: 17A2, BioLegend, Cat#100227): use of antibody for FC (1:1000 dilution) was validated by BioLegend.
13. Rat anti-mouse CD19-APC (clone: 6D5, BioLegend, Cat#115512): use of antibody for FC (1:1000 dilution) was validated by BioLegend.
14. AlexaFluor 488-labeled alpha-bungarotoxin (Invitrogen, Cat#B13422): use of alpha-bungarotoxin for IHC (1 μg/mL) by Invivotek LLC (Hamilton, NJ).
15. Donkey anti-mouse IgG-HRP (abcam, Cat#ab7061): use of antibody for ELISA (1:20,000-1:80,000 dilution) was validated by abcam.
16. Goat anti-Mouse IgG1 (Cat#1071-05), IgG2b (Cat#1090-05), IgG2c (Cat#1078-05), and IgG3 (Cat#1101-05) from SouthernBiotech: use of antibodies for ELISA (1:4,000-1:8,000 dilution) was validated by SouthernBiotech.
17. Mouse anti-human IgG-PE (BD Biosciences, Cat#555787): use of antibody for FC (1:1000 dilution) was validated by BD Biosciences.
18. Mouse anti-human IgG4 Fc-AF647 (SouthernBiotech, Cat#9200-31): use of antibody for FC (1:1000 dilution) was validated by SouthernBiotech.
19. PE-labeled Human CD19 (20-291) protein (ACROBiosystems, Cat#CD9-HP2H3): use of conjugated protein for FC (1:50 dilution) was validated by ACROBiosystems.

Eukaryotic cell lines

Policy information about cell lines

Cell line source(s)	Lenti-X 293T cells were purchased from Takara (Cat#632180). U87-MG cells were purchased from ATCC (Cat#HTB14Luc2). C2C12 cells were purchased from ATCC (Cat#70024392). Primary human cells: skeletal muscle cells (ZenBio, Cat# SKB-F), iPSC-derived iCell cardiomyocytes and iPSC-derived iCell GANAneurons (Fuji Cellular Dynamics, Cat#R1007/R1106 and R1013/R1011); HBEC (Lonza, Cat#CC-2540); primary hepatocytes and HREC (InnoProt, Cat#P10651 and #P10664); NHEM (Lonza, Cat#CC-2586 and LifeLine, Cat#FC-0030); HUVEC (Lonza, Cat#CC-2517); peripheral blood monocytes (StemCell technologies, Cat#200-0067); peripheral blood NK cells (StemCell technologies, Cat#200-0064).
Authentication	Nalm-6 cells and Jurkat cells were periodically authenticated by flow cytometry after staining with either anti-CD19 or anti-CD3 antibody. The other cell lines are not authenticated since they are shortly cultured during each experiment.
Mycoplasma contamination	All cells were periodically tested for mycoplasma contamination. None of the cell lines were contaminated.
Commonly misidentified lines (See ICLAC register)	No commonly misidentified cell lines were used in this study

Animals and other organisms

Policy information about studies involving animals; ARRIVE guidelines recommended for reporting animal research

Laboratory animals	NSG mice (male, 6-8 weeks) were provided by the SCXC core at the University of Pennsylvania. CD45.1 and CD45.2 C57BL/6J mice (6-8 weeks, male) were purchased from Jackson Laboratories
Wild animals	No wild animals were used in this study
Field-collected samples	No field-collected samples were used in this study
Ethics oversight	All studies involving animals were performed under a protocol approved by the University of Pennsylvania Institutional Animal Care and Use Committee.

Note that full information on the approval of the study protocol must also be provided in the manuscript.

Human research participants

Policy information about studies involving human research participants

Population characteristics	*Describe the covariate-relevant population characteristics of the human research participants (e.g. age, gender, genotypic information, past and current diagnosis and treatment categories). If you filled out the behavioural & social sciences study design questions and have nothing to add here, write "See above."*
Recruitment	*Describe how participants were recruited. Outline any potential self-selection bias or other biases that may be present and how these are likely to impact results.*
Ethics oversight	*Identify the organization(s) that approved the study protocol.*

Note that full information on the approval of the study protocol must also be provided in the manuscript.

Flow Cytometry

Plots

Confirm that:

☒ The axis labels state the marker and fluorochrome used (e.g. CD4-FITC).

☒ The axis scales are clearly visible. Include numbers along axes only for bottom left plot of group (a 'group' is an analysis of identical markers).

☒ All plots are contour plots with outliers or pseudocolor plots.

☒ A numerical value for number of cells or percentage (with statistics) is provided.

Methodology

Sample preparation	Lymphocytes were isolated from cranial bone marrow using a previously reported protocol6. In brief, the calvaria was cut into small pieces using sterile scissors and dissociated in PBS + 2% FBS with a pestle. Spleens were harvested from mice, washed in PBS, and cut into 0.5 mm cubes in ice-cold PBS. Spleen or bone marrow isolates were transferred to a 70 µm cell strainer (Falcon, Cat#352350); cells were washed with PBS and resuspended in RBC lysis buffer (BioLegend, Cat#420301). Cells were stained for 30 minutes on ice using the following antibodies: anti-CD3 BV711 or anti-CD3-AF647 (clone: okt3, BD Biosciences, Cat#750983 and Cat#566686)), anti-MuSK PE (clone: 189-1 or 24C10), anti-human Ig light chain lambda PE (Clone: MHL-38, BioLegend, Cat#316608), anti-human Ig light chain kappa APC (clone: MHK-49, BioLegend, Cat#316510), anti-human IgG PE (BD Biosciences, Cat#555787), and/or anti-mouse IgG APC (clone A85-1, BD Biosciences).
Instrument	BD LSR II flow cytometer
Software	FlowJo (Tree Star, V10.8.1)
Cell population abundance	The purity of CD45.1 mouse T cells was >95% after sorting (R&D Systems, Cat#MTCC-25).
Gating strategy	Figure 1b: gating for positive cells was selected less than 0.3% in negative control cells.<br>Figure 2d: CTV plots are shown after gating singlets (FSC-A/FSC-H/FSC-W/SSC-W), lymphocytes (SSC-A/FSC-A), and live/dead staining (gating strategy is shown in Extended data Fig. 4c).<br>Figure 4a: CD45.2 positive cells are shown after singlet and lymphocyte gating.

☒ Tick this box to confirm that a figure exemplifying the gating strategy is provided in the Supplementary Information.
